# A Comprehensive Course for Teaching Emergency Cricothyrotomy

**DOI:** 10.21980/J8JS9W

**Published:** 2020-01-15

**Authors:** Brandon Backlund, Richard Utarnachitt, Joshua Jauregui, Taketo Watase

**Affiliations:** * University of Washington, Department of Emergency Medicine, Seattle, WA

## Abstract

**Audience:**

This curriculum was developed for emergency medicine (EM) residents at the post-graduate year (PGY) 1–4 level, and attending EM physicians. It may be adapted for training of any healthcare provider or learner who might be required to perform an emergency cricothyrotomy, including emergency medical technicians, senior medical students, and advanced practice providers (ie, nurse practitioners and physician assistants); however, we did not specifically validate it for these providers.

**Introduction:**

Emergency cricothyrotomy (EC) is a lifesaving surgical procedure, often the option of last resort, used to secure the airway when other methods of airway control have failed or are not feasible. It is a high-risk procedure since it is infrequently performed, but it is time-sensitive and critical for survival when needed.^1^,^2^ Time-sensitive procedural skills such as EC are subject to relatively rapid decay,^3^,^4^ and unlike other high-risk procedures, in which just-in-time training (JITT) may improve real time procedural performance, the extreme time sensitivity of cricothryotomy precludes JITT as a feasible educational intervention to improve EC performance.^5^ As such, clinicians must periodically review the essential concepts and practice the physical actions of the procedure in order to build and maintain familiarity with the steps involved and to develop and maintain the muscle memory necessary to perform it quickly and confidently. Previous studies have shown that simulation-based training improves both confidence and competence in the performance of the simulated procedures,^6^,^7^ and that small group learning situations are effective for procedural learning.^8^,^9^

Commercially produced mannequins are available to simulate cricothyrotomy. However, being made of plastic materials, they suffer from unrealistic “tissue” feel that is radically different from that of biologic tissue.^10^,^11^ Additionally, because they are mass-produced, they tend to be fairly homogeneous in their anatomic representations, lacking the variability encountered in the human population.

We developed an inexpensive procedure simulator using commercially available porcine byproduct that more closely mimics the feel of cricothyrotomy in real life, and a comprehensive curriculum for instruction in, or review of, EC, intended for implementation in a small-group format. This publication is intended to provide interested educators with a comprehensive suite of materials for teaching EC at their own institution. Included are instructions for how to construct the simulator, an EC case scenario with discussion points, a PowerPoint didactic module covering the fundamental concepts of EC, and sample course evaluation forms that may be implemented directly or adapted to meet institutional requirements.

**Educational Objectives:**

After completing this activity, the learner will be able to:

1. correctly describe the indications for and contraindications to emergency cricothyrotomy
2. correctly describe and identify on the simulator the anatomic landmarks involved in emergency cricothyrotomy
3. correctly list the required equipment and the sequence of the steps for the “standard” and “minimalist” variations of the procedure
4. demonstrate proper technique when performing a cricothyrotomy on the simulator without prompts or pauses

**Educational Methods:**

Small group activity combining didactic learning, case-based learning, and procedural simulation. The didactic component may be delivered in an asynchronous learning or “flipped classroom” format.

**Research Methods:**

The cricothyrotomy simulator was initially pilot-tested on a group of emergency medicine attending faculty, who were asked to evaluate the simulator, with results demonstrating that it was felt to be superior to typical plastic mannequin simulators. This simulator was then subsequently integrated into our airway workshops teaching EC, which include hands-on practice, didactic, and discussion components. The content and delivery of these workshops were assessed by the learners via standardized evaluation forms after completion of each workshop, and the overall clinical relevance, appropriateness of content, and satisfaction with the workshop format were highly rated (mean score 4.87 on a 5-point scale, with 5 denoted as “Excellent”).

**Discussion:**

The real-tissue model to simulate the procedure was well liked by course participants, and the learning environment was felt to be conducive to asking questions and discussion. Overall, the instructors and the learners felt that the workshops were effective in improving understanding of the procedure and increasing the comfort level and skill of the emergency physician learners in performing the procedure.

**Topics:**

Cricothyrotomy, cricothyroidotomy, emergency airway, surgical airway, failed airway, rescue airway, can’t intubate can’t ventilate, small group activity, simulation.

## USER GUIDE

[Table t1-jetem-5-1-sg17]

**Table t1-jetem-5-1-sg17:** 

List of Resources:	
Abstract	17
User Guide	19
Small Groups Learning Materials	22
Appendix A: Cricothyrotomy Simulator Assembly Instructions	22
Appendix B: Case Scenario with Instructor Prompts and Discussion Points	28
Appendix C: Case Scenario Handout	30
Appendix D: Emergency Cricothyrotomy PowerPoint without Case Scenario	32
Appendix E: Emergency Cricothyrotomy PowerPoint with Case Scenario	33
Appendix F: Emergency Cricothyrotomy Course Evaluation Form	34
Appendix G: Emergency Cricothyrotomy Quick Reference	35

**Learner Audience:**

Medical Students, Interns, Junior Residents, Senior Residents, Fellows and Faculty

**Time Required for Implementation:**

Approximately 1 hour:

1. Presentation and discussion of “Emergency Cricothyrotomy Case Scenario” (15 minutes)
2. Presentation of “Emergency Cricothyroidotomy” PowerPoint slides, questions and discussion (20 minutes, may be done asynchronously)
3. Hands-on practice of emergency cricothyrotomy procedure on real-tissue simulator (5 minutes per learner)
4. Questions, discussion, and debrief, complete evaluations (10–15 minutes)

**Recommended Number of Learners per Instructor**:

Maximum of 4 learners per instructor

**Topics:**

Cricothyrotomy, cricothyroidotomy, emergency airway, surgical airway, failed airway, rescue airway, can’t intubate can’t ventilate, small group activity, simulation.

**Objectives:**

After completing this course, the learner should be able to:

1. Correctly describe the indications for and contraindications to emergency cricothyrotomy
2. Correctly describe and identify on the simulator the anatomic landmarks involved in emergency cricothyrotomy
3. Correctly list the required equipment and the sequence of the steps for the “standard” and “minimalist” variations of the procedure
4. Demonstrate proper technique when performing a cricothyrotomy on the simulator without prompts or pauses

### Linked objectives and methods

The components of this course are designed to provide flexibility for adaptation to different education strategies, learning environments, and group sizes. Included components comprise a case-based learning module, a didactic educational module, and a practical or “hands-on” training session on a simulator. We believe that it is important for the learner to be exposed to all three elements in order to develop or reinforce the required theoretical, practical, and integrative knowledge essential to effective performance of EC.

1. Case-based learning and discussion: an example case of an airway emergency secondary to upper airway obstruction is presented, with planned pauses for learner-driven discussion, facilitated by the instructor, and prompted by questions embedded in the case. This component may be deployed as a stand-alone discussion, or it may be integrated with the educational module (see #2 below).
2. Educational module: a PowerPoint slide set covering the essential concepts underlying EC, including indications and contraindications to the procedure (objective 1), relevant anatomy, necessary equipment, and the technical steps in sequence of the procedure (objective 3). This component may be assigned for review prior to the course, using an asynchronous learning or “flipped classroom” model, or it may be integrated into a classroom-style didactic lecture in combination with element #1 above. This allows for flexibility to accommodate different learning styles, time constraints, and teaching environments.
3. Practical training sessions (objectives 2 and 4): we developed a model to simulate cricothyrotomy, using skin and the larynges with attached tracheae from butchered pigs, obtained via a commercial vendor. Previous studies and our own evaluation data have suggested that simulators for EC that use biologic tissues, such as our model, are felt to provide a more realistic simulation of the procedure than those that use plastic or other synthetic materials.^10^,^11^ Detailed instructions on preparing the simulator that we developed are included in Appendix A.

### Recommended pre-reading for facilitator

- The instructor should review the “Emergency Cricothyrotomy” PowerPoint file and the case scenario prior to the workshop in order to ensure familiarity with the content to which the learners will be exposed and around which discussion will be generated.
- The instructor should be familiar and comfortable with teaching and demonstrating EC, but no other specific preparation is otherwise required.

### Learner responsible content (LRC)

- If a “flipped classroom” model is to be employed, the learners should be given access to the “Emergency Cricothyrotomy” PowerPoint file for viewing prior to the workshop. The file size (3MB) is small enough to be attached to most common email providers, such as Outlook and Gmail, or it may be made available via a cloud storage server for download.

### Small group application exercise (sGAE)

NOTE: Two versions of this sGAE are included in the Supplemental Materials:

- Appendix B, with instructor prompts and discussion points, intended for use by the facilitator/instructor; and Appendix C, a version without the instructor prompts, which may be used as a handout if desired.
- Additionally, two versions of the “Emergency Cricothyrotomy” PowerPoint file are included, Appendix D, without the case scenario, if the module has been assigned for asynchronous review prior to the course, and Appendix E, which includes the case scenario, if the case scenario and the didactic module are to be delivered by the instructor as a unit.

### Results and tips for successful implementation

We developed a simulator for performing EC using pig tracheas and skin (assembly instructions are included in Appendix A). This simulator was initially pilot-tested on a group of 10 Emergency Medicine (EM) attending faculty members, who performed a cricothyrotomy on the simulator and were subsequently asked to evaluate the pig model to assess their impression of the fidelity of the model, and their opinion of the model compared to a standard plastic mannequin. They were asked to assess the pig model using two questions; Question 1 asked, “Compared to a mannequin cric [sic] model, was the pig cric model realistic?” Answer options were on a 5-point scale, with 1 being “extremely unrealistic” and 5 being “extremely realistic”; for this question, the mean was 4.4 (SD 0.7). Question 2 asked, “In future cric training, would you like to use the pig cric model or mannequin cric model?” One hundred per cent of participants indicated a preference for the pig model.

Based on these favorable assessments of the simulator, it was subsequently integrated into our EC workshops, in which the fundamental concepts of EC were taught, including case scenarios to prompt discussion and thought. An example case scenario and a PowerPoint didactic module are included as Appendix B and Appendix C, respectively. The workshop in this format was completed by a total of 64 learners, including EM residents (n=24) and faculty (n=42), on four separate occasions. Following completion of the course, participants were asked to assess its content and delivery via a standard anonymous evaluation form on a 5-point scale, with 1 being “Poor” and 5 being “Excellent”; overall, the average summative rating was 4.87 (SD 0.3).

As with most procedures, successful performance of EC requires both theoretical and technical knowledge about the procedure. We feel that it is important that the learner understands the fundamental underlying concepts, including indications, contraindications, relevant anatomy, and potential complications, as well as having the opportunity to practice the sequence and physical actions of the procedure itself.

Depending on the institutional preferences and available time, the “Emergency Cricothyrotomy” PowerPoint presentation may be made available for viewing by the learner audience before the course (asynchronous or “flipped classroom” approach), with the case-based discussion and procedure practice on the simulator occurring on the day of the workshop.

Alternatively, the “Emergency Cricothyrotomy” PowerPoint presentation may be combined with the case-based discussion in a classic lecture format; the recommended sequence in this scenario would be: 1. Case-based discussion; 2. “Emergency Cricothyrotomy” PowerPoint presentation; 3. Cricothyrotomy simulator practicum.

### Pearls

This is included separately as Appendix G, “Emergency Cricothyrotomy Quick Reference” Word document, which can be printed out and distributed as a handout after the course.)

## Supplementary Information



## Figures and Tables

**Figure 1:**
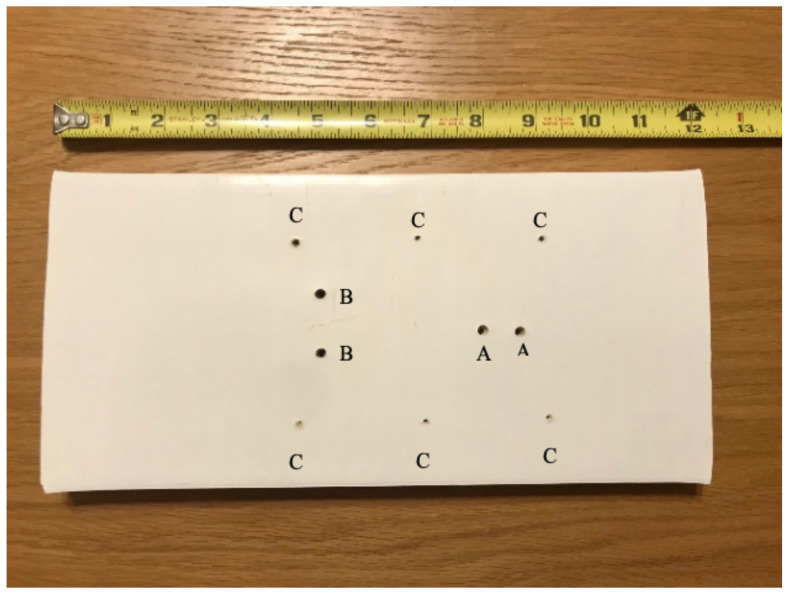
base board showing placement of pre-drilled holes

**Figure 2:**
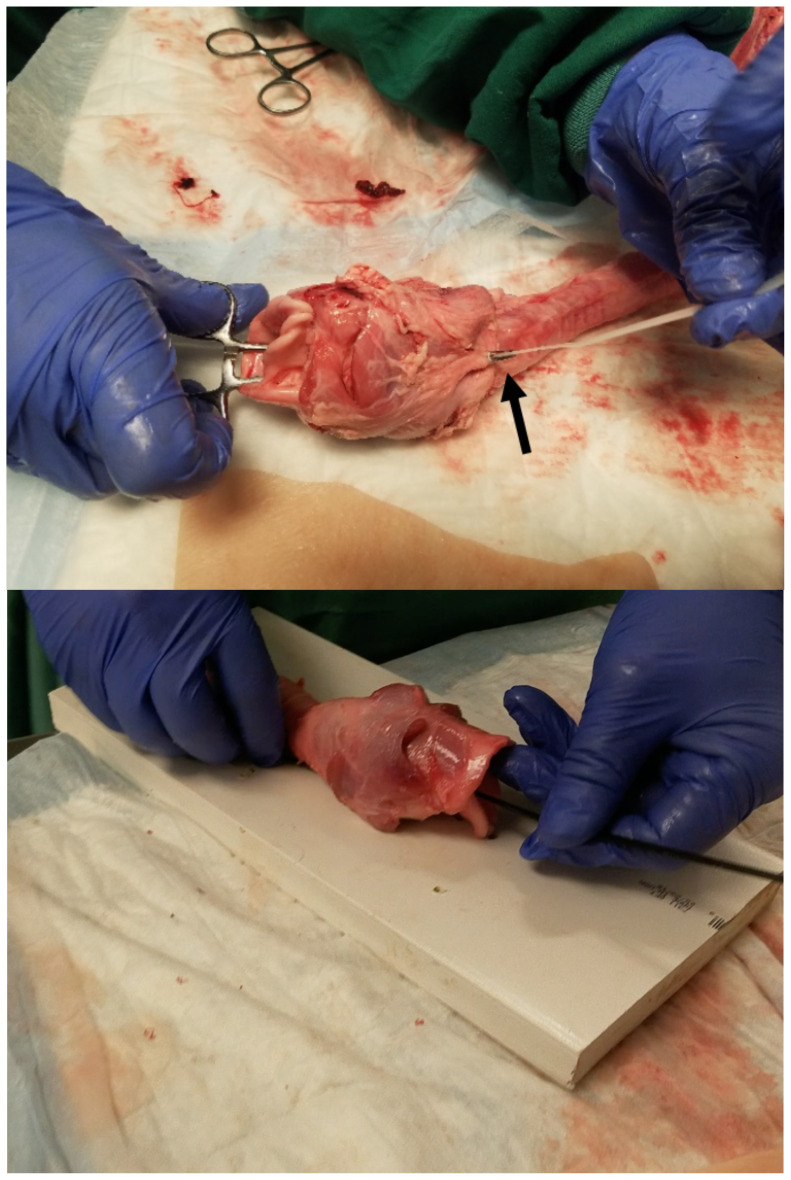
Use clamp to grab zip tie and thread through larynx. Black arrow shows tip of hemostat protruding through posterior trachea and gripping tip of zip tie.

**Figure 3:**
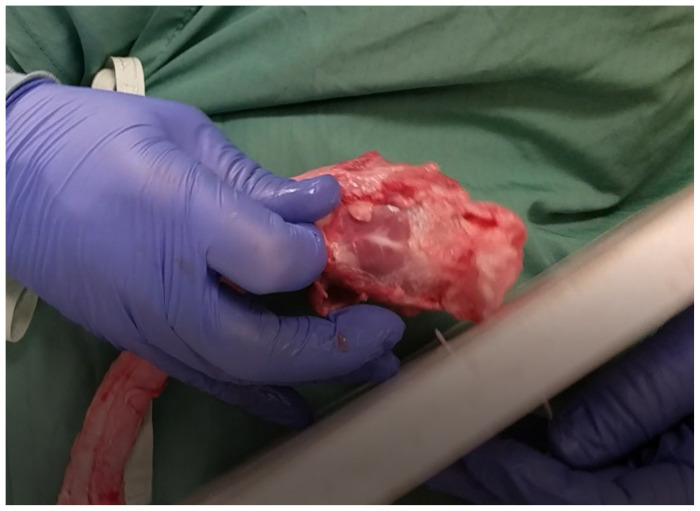
insert zip tie through holes in board

**Figure 4:**
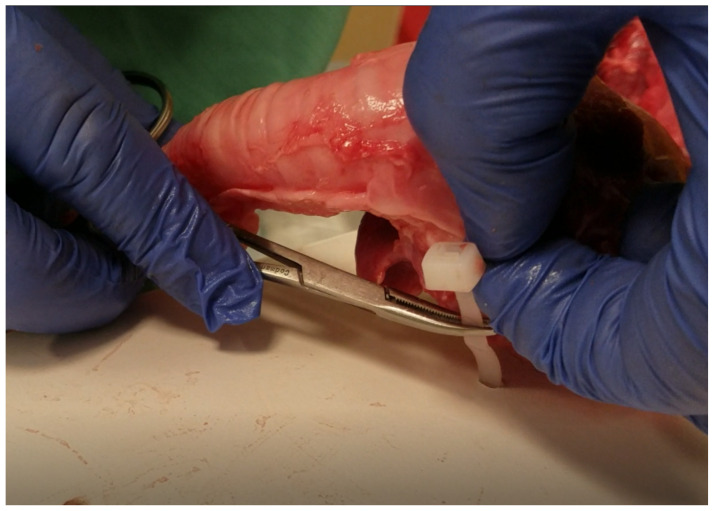
secure pig trachea to the base board with zip tie

**Figure 5:**
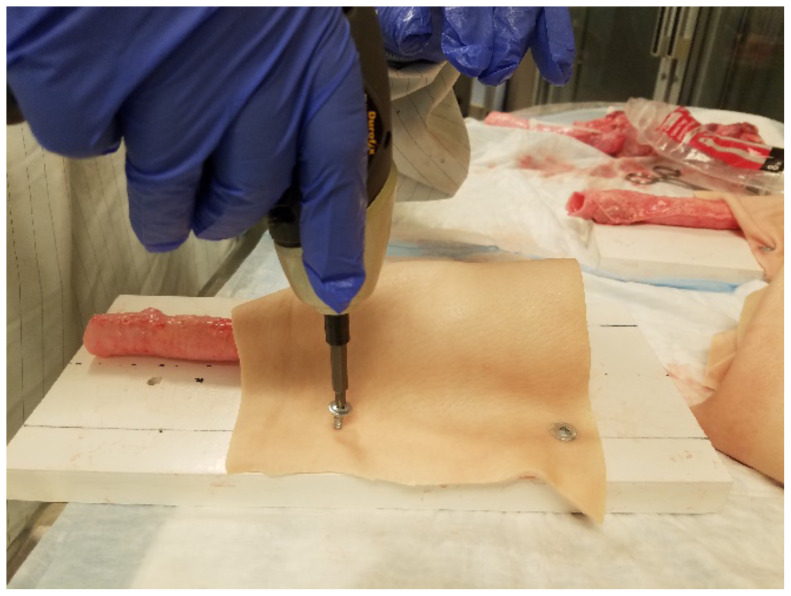
drilling skin to the base board

**Figure 6:**
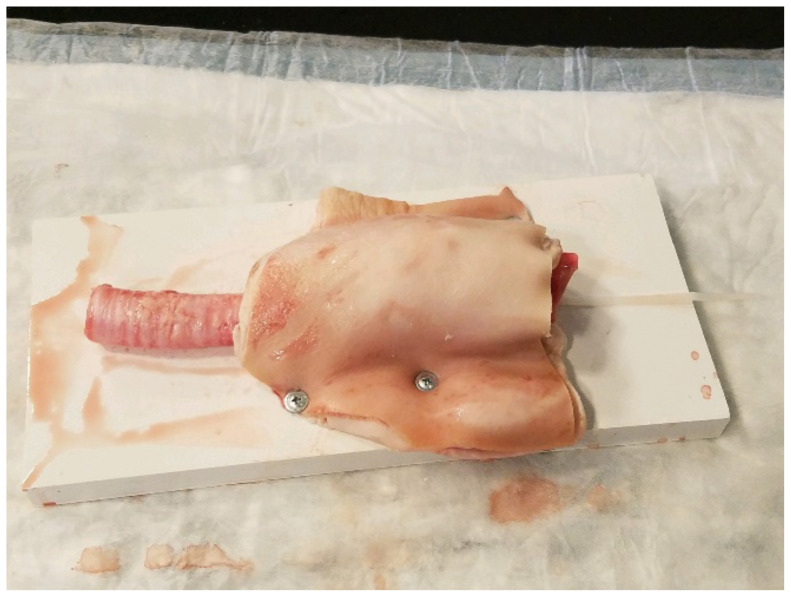
finished model ready for use

## SMALL GROUPS LEARNING MATERIALS

### Appendix A Cricothyrotomy Simulator Assembly Instructions

This model can be fabricated using easily obtained materials from any local hardware store. A supplier for pig skin and pig trachea must also be secured. Since this is not always available at the local grocery store or butcher, we used a commercial vendor (Clemens Food Group, Hatfield, PA, https://clemensfoodgroup.com), who delivered the tracheas frozen and pre-cut to an approximate length of 10 inches (in.); the skin was delivered in sheets measuring approximately 12 × 12 in.

#### List of required items

[Table t2-jetem-5-1-sg17]

**Table t2-jetem-5-1-sg17:**

Item	Quantity needed
1. 1 × 6 inch PVC board, cut to length of 12 in.	1 per learner
2. Electric or battery-powered hand drill 1/8” drill bit 1/4” drill bit Philips head screwdriver bit	1 of each
3. Phillips truss head self-piercing lath screws, size #8 × 9/16 in. length	6 per base unit
4. Plastic zip ties, 10–12 in. length	2 per base unit
5. #11 blade scalpel	1
6. Pig airway (including larynx and trachea)	1
7. Pig skin	1
8. Curved clamp/hemostat (Kelly, mosquito, or similar)	1

#### Approximate cost of items

[Table t3-jetem-5-1-sg17]

**Table t3-jetem-5-1-sg17:**

• Each trachea/larynx unit, including skin:	$5
• Zip ties:	$0.20 each
• *Board base:	$4 apiece
• *Screws:	$0.50 for 6
• *Electric screwdriver:	$20

* Reusable items, one-time cost

Because the base, screws, and screwdriver are reusable, the cost per learner to use the simulator subsequently is therefore only approximately $5.

#### Detailed methods for construction

##### Step 1: Preparing the base board

Start by obtaining 1 × 6 in. PVC board from a hardware supply store, cut into 12 in. lengths. To prepare the board base: (Figure 1)

Use a tape measure to mark out the holes for drilling. Measuring from the bottom of the board lengthwise along the centerline, use the 1/4″ drill bit to drill two vertical holes (labeled “A” in Figure 1) completely through the board, one at 9 in. and the second one at 8 in. Two more horizonal holes (labeled “B” in Figure 1) should be drilled, again using the 1/4″ bit, completely through the board at approximately 5 in., one on either side of the centerline, spaced about 1 to 1.5 in. apart. These holes are used to pass the zip ties through to secure the pig trachea to the base board. Next use the drill with the 1/8″ bit to make three holes (labeled “C” in Figure 1) on both sides of your original set of holes. Drill these only to the depth needed to accommodate the length of the screws, about ½ inch; they do not need to traverse the full thickness of the board. These superficial holes will be used to help guide the screws when securing the pig skin over the trachea.

##### Step 2: Securing the trachea to the board

Taking the pig trachea, make a small hole using either a scalpel or small scissors to the posterior trachea just where it joins the posterior larynx. Take a Kelly clamp or needle driver, insert it into the vestibule of the superior larynx, through the hole just made in the posterior trachea, and grasp the zip tie with the clamp. Pull the zip tie through this hole and back out the laryngeal vestibule (Figure 2).

Thread the zip tie through the centrally located vertical holes in the base board (Figure 3, Video)

Secure the zip tie so that the posterior aspect of the thyroid cartilage is flush to the board (Figure 4). Snip off the excess length of the end of the zip tie. (***Optional.)*** Take another zip tie, and secure the trachea to the centrally-located horizontal holes in a similar fashion.

##### Step 3: Preparing the pig skin

Pig skin comes in large sheets from the supplier. Trim the skin to a size that will completely cover the structures of the thyroid cartilage, cricothyroid membrane, and cricothyroid cartilage, and most of the remaining trachea. A #11 blade scalpel is useful for cutting the pig skin to size.

Be sure to leave enough skin on the sides so that the Philips piercing screws will be able to affix the skin to the board (Figure 5).

##### Step 4: Securing skin over the pig airway and to base

Utilize an appropriately sized pig skin that will cover the pig trachea and at least most of the remaining thyroid cartilage. This will simulate skin over the cricothyroid membrane, so it should be somewhat taut over the underlying structures. Use the hand drill to affix the truss screws through the skin and into the lateral holes that were drilled in the previous steps into the base board (Figure 5). Depending on the thickness of the skin, it may be necessary to make holes in the skin to assist drilling the screws through the skin and into the board. Figure 6 represents a finished model ready for use.

#### Tips for Implementation

- Ideally, assembly should take place prior to the actual session to allow ample time to prepare an adequate number of models for the number of leaners participating.
- It takes approximately 5 minutes to construct each base unit from scratch, but once built, it only takes approximately 1–2 minutes to replace a new skin/larynx assembly onto the base after the previous one has been used.
- The pig parts may be kept frozen, and thawed 1–2 days prior to the course for preparation. We found that the tissues had no change in texture and feeling after freezing and thawing.
- To facilitate swift changeover after the model has been used, we found that inserting the zip ties through the larynx assembly ahead of time made it much easier to replace a new one on the base to prepare the model for the next learner.
- The tissue components should be disposed of after use in a receptacle appropriate for biologic waste.
- In between courses, we recommend washing the base unit and screws with soap & water, and wiping down with a disinfectant before storage.
- We used board for the base made of synthetic material (PVC), because it is nonporous and does not absorb biologic fluids. Wood board could be used, but would be difficult to clean after use and might degrade over time.

### Appendix B Case Scenario with Instructor Prompts and Discussion Points

#### Chief Complaint: Respiratory Distress

History of present illness: 47-year-old male brought to the emergency department by ambulance for difficulty breathing. The patient has a limited ability to speak since he is very agitated, but does endorse heavy crack cocaine use over the past several days.

**Allergies:** None

**Medications:** “Something for blood pressure”

**Past Medical History**: Hypertension, polysubstance use

**Social History**: +Smokes crystalline cocaine (crack), +smokes tobacco, + daily alcohol use

**Review of systems:** Unable to obtain due to patient being in extremis

***Pertinent Physical Exam:***

**Vitals:** Heart rate (HR) 122, blood pressure (BP) 180/110, temperature (T) 37°C, respiratory rate (RR) 34, oxygen saturation (0_2_sat) 88%

**General:** appears to be in distress, sitting bolt upright in gurney, agitated and diaphoretic

**Head, eyes, ears, nose and throat (HEENT):** no obvious mass in mouth, mucus membranes dry, prior tracheostomy scar noted, voice hoarse and muffled

**Cardiovascular:** tachycardic

**Lungs:** coarse audible upper airway sounds, inspiratory stridor, supraclavicular retractions

**Abdomen:** soft

**Extremities:** radial pulse present, no lower extremity edema

**Skin:** Warm, pale, profusely diaphoretic

**Pause.** Please turn to a person next to you and discuss the following question for 2 minutes:

**What are your concerns and considerations regarding this patient’s airway and the need for airway management?**

**Instructor: Facilitate summary of discussions, emphasizing the following aspects of the case:**

1. **Identification of respiratory distress**
2. **Identification of tachypnea, hypoxia, and potential impending respiratory failure**
3. **Concern for possibility of upper airway obstruction**
4. **Potential for difficult airway**
5. **What airway equipment should be gathered at this point?**
  - **Non-invasive positive pressure ventilation, eg, bilevel positive airway pressure (BiPAP), may not be tolerated**
  - **Supraglottic airway, eg, iGel, laryngeal mask airway (LMA) placement may not be possible given possible upper airway obstruction**
  - **Difficult airway equipment should be made readily available in addition to standard airway management**

**Continue with case:**

The emergency medicine physician anticipates a potentially difficult airway; therefore, as the patient is being placed on the monitor, a cricothyrotomy tray is brought to the bedside as well as video laryngoscopy (VL) set up and an appropriately sized laryngeal mask airway (LMA).

The patient’s oxygen saturation is in the 80s despite being on a non-rebreather mask and a nasal canula at flush rate greater than 15 liters/minute. The patient is agitated, pulling at the facemask, and unable to tolerate assisted ventilation with bag valve mask (BVM). His oxygen saturation now drops into the 70s, and he is starting to become more obtunded. Etomidate 20 milligrams (mg) and succinylcholine 150 mg are administered intravenously (IV). The emergency medicine physician attempts to intubate with VL and sees significant swelling in the posterior oropharynx and a large soft tissue mass obstructing the view of the glottic opening.

**Pause.** Please turn to a person next to you and discuss the following question for 2 minutes:

**How do you approach the next step in airway management?**

**Instructor: Facilitate summary of discussions, emphasizing the following aspects of the case:**

1. **Inability to oxygenate by non-invasive means, critically low oxygen saturation**
2. **Evidence of ineffective ventilations (agitation, hypoxia)**
3. **Evidence of upper airway obstruction, unable to visualize glottis**
4. **Impending “can’t ventilate, can’t intubate” scenario--need to prepare for rescue or surgical airway**

**Break for Questions**

### Appendix C Case Scenario Handout

#### Chief Complaint: Respiratory Distress

History of present illness: 47-year-old male brought to the emergency department by ambulance for difficulty breathing. The patient has a limited ability to speak since he is very agitated, but does endorse heavy crack cocaine use over the past several days.

**Allergies:** None

**Medications:** “Something for blood pressure”

**Past Medical History**: Hypertension, polysubstance use

**Social History**: +Smokes crystalline cocaine (crack), +smokes tobacco, + daily alcohol use

**Review of systems:** Unable to obtain due to patient being in extremis

***Pertinent Physical Exam:***

**Vitals:** Heart rate (HR) 122, blood pressure (BP) 180/110, temperature (T) 37°C, respiratory rate (RR) 34, oxygen saturation (0_2_sat) 88%

**General:** appears to be in distress, sitting bolt upright in gurney, agitated and diaphoretic

**Head, eyes, ears, nose and throat (HEENT):** no obvious mass in mouth, mucus membranes dry, prior tracheostomy scar noted, voice hoarse and muffled

**Cardiovascular:** tachycardic

**Lungs:** coarse audible upper airway sounds, inspiratory stridor, supraclavicular retractions

**Abdomen:** soft

**Extremities:** radial pulse present, no lower extremity edema

**Skin:** Warm, pale, profusely diaphoretic

**Pause.** Please turn to a person next to you and discuss the following question for 2 minutes:

**What are your concerns and considerations regarding this patient’s airway and the need for airway management?**

**Continue with case:**

The emergency medicine physician anticipates a potentially difficult airway; therefore, as the patient is being placed on the monitor, a cricothyrotomy tray is brought to the bedside as well as video laryngoscopy (VL) set up and an appropriately sized laryngeal mask airway (LMA).

The patient’s oxygen saturation is in the 80s despite being on a non-rebreather mask and a nasal canula at flush rate greater than 15 liters/minute. The patient is agitated, pulling at the facemask, and unable to tolerate assisted ventilation with bag valve mask (BVM). His oxygen saturation now drops into the 70s, and he is starting to become more obtunded. Etomidate 20 milligrams (mg) and succinylcholine 150 mg are administered intravenously (IV). The emergency medicine physician attempts to intubate with VL and sees significant swelling in the posterior oropharynx and a large soft tissue mass obstructing the view of the glottic opening.

**Pause.** Please turn to a person next to you and discuss the following question for 2 minutes:

**How do you approach the next step in airway management?**

### Appendix D Emergency Cricothyrotomy PowerPoint without Case Scenario

PowerPoint file if the module has been assigned for asynchronous review prior to the course.

### Appendix E Emergency Cricothyrotomy PowerPoint with Case Scenario

PowerPoint file if the case scenario and the didactic module are to be delivered by the instructor as a unit.

### Appendix F Emergency Cricothyrotomy Course Evaluation Form

[Table t4-jetem-5-1-sg17]

**Table t4-jetem-5-1-sg17:**

Workshop Evaluation: Emergency Cricothyrotomy	
Date of Workshop:	
Instructor:	

***Please respond to the following statements using the 5-point rating scale below. Please circle the number that best describes your response.*** [Table t5-jetem-5-1-sg17]

**Table t5-jetem-5-1-sg17:**

1. How clear were the objectives to you?				
1-Not at all	2-Slightly	3-Moderately	4-Quite a bit	5-A Tremendous Amount
2. Were the workshop objectives met?				
1-Not at all	2-Slightly	3-Moderately	4-Quite a bit	5-A Tremendous Amount
3. How well-organized was the workshop?				
1-Not at all	2-Slightly	3-Moderately	4-Quite a bit	5-A Tremendous Amount
4. Was the information provided and skills covered relevant and useful?				
1-Not at all	2-Slightly	3-Moderately	4-Quite a bit	5-A Tremendous Amount
5. Did the instructor teach the workshop effectively?				
1-Not at all	2-Slightly	3-Moderately	4-Quite a bit	5-A Tremendous Amount
6. Was the cricothyrotomy simulator an effective model on which to practice the procedure?				
1-Not at all	2-Slightly	3-Moderately	4-Quite a bit	5-A Tremendous Amount
7. Do you feel more confident in your ability to perform an emergency cricothyrotomy after completing the workshop?				
1-Not at all	2-Slightly	3-Moderately	4-Quite a bit	5-A Tremendous Amount
8. Was there adequate time provided for questions and discussion?				
1-Not at all	2-Slightly	3-Moderately	4-Quite a bit	5-A Tremendous Amount
9. Was the workshop free of commercial bias?				
1-Not at all	2-Slightly	3-Moderately	4-Quite a bit	5-A Tremendous Amount

***Comments or Suggestions:***

### Appendix G Emergency Cricothyrotomy Quick Reference

#### Indications

Emergency airway required, and:

- Failed orotracheal or nasotracheal intubation
- Rescue airway attempts failed or not feasible
- Unable to adequately oxygenate and/or ventilate patient
- “Can’t ventilate, can’t intubate”

#### Contraindications

- Tracheal transection
- Direct trauma to laryngeal area obscuring or damaging relevant anatomic area and landmarks
- Obstruction distal to cricothyroid membrane
- Age <10 years (relative contraindication)

#### Equipment

[Table t6-jetem-5-1-sg17]

**Table t6-jetem-5-1-sg17:**

**Standard Technique** Personal protective equipment (PPE) Skin disinfectant (e.g. Betadine, chlorhexidine) Suction Scalpel Cuffed tracheostomy tube (eg, #4 Shiley) 10mL syringe Tracheal hook Tracheal dilator (Optional) elastic bougie	**Minimalist Technique** PPE Skin disinfectant Suction Scalpel 6-0 cuffed endotracheal tube 10mL syringe Elastic bougie
