# Supplementary material for: A Comprehensive Course for Teaching Emergency Cricothyrotomy
Source: J Educ Teach Emerg Med. 2020 Jan 15;5(1):SG17–35. doi: 10.21980/J8JS9W (PMC10332533; doi:10.21980/J8JS9W)
Supplement: Supplementary file 2 — PowerPoint file if the module has been assigned for asynchronous review prior to the course. [file jetem-5-1-sg17-appendixd.pptx]

## Slide 1
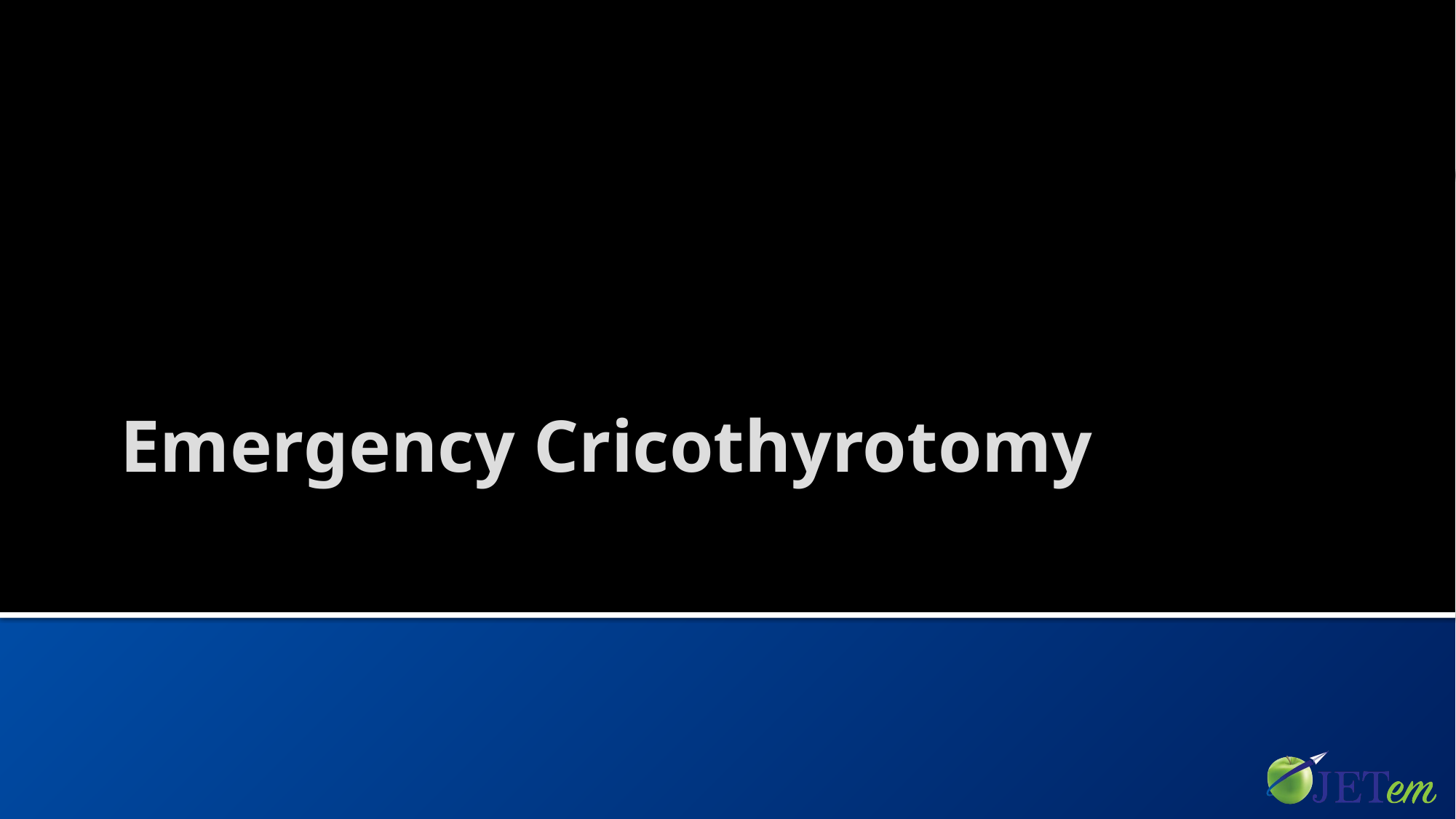

# Emergency Cricothyrotomy

## Slide 2
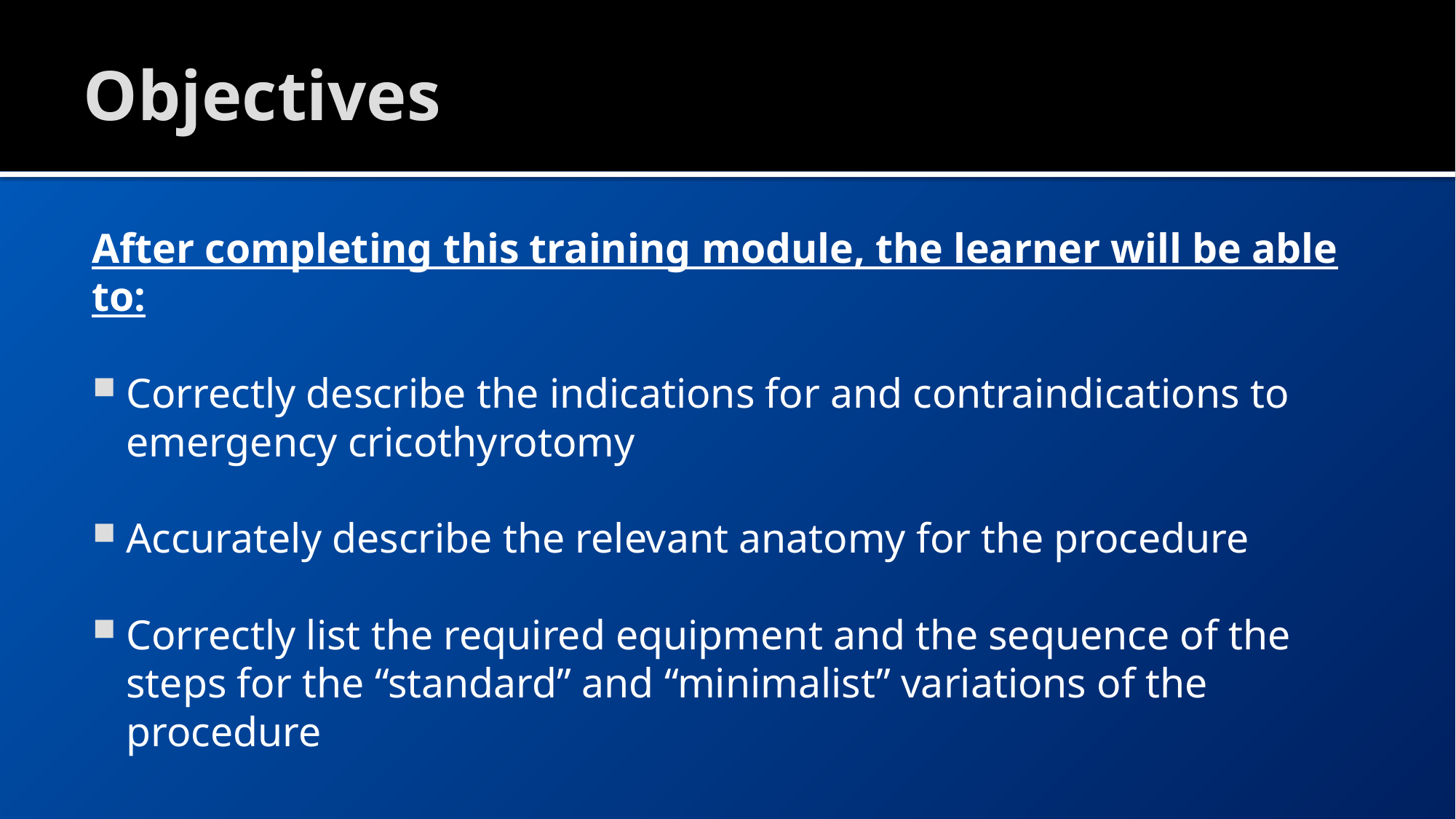

# Objectives
After completing this training module, the learner will be able to:
Correctly describe the indications for and contraindications to emergency cricothyrotomy
Accurately describe the relevant anatomy for the procedure
Correctly list the required equipment and the sequence of the steps for the “standard” and “minimalist” variations of the procedure

## Slide 3
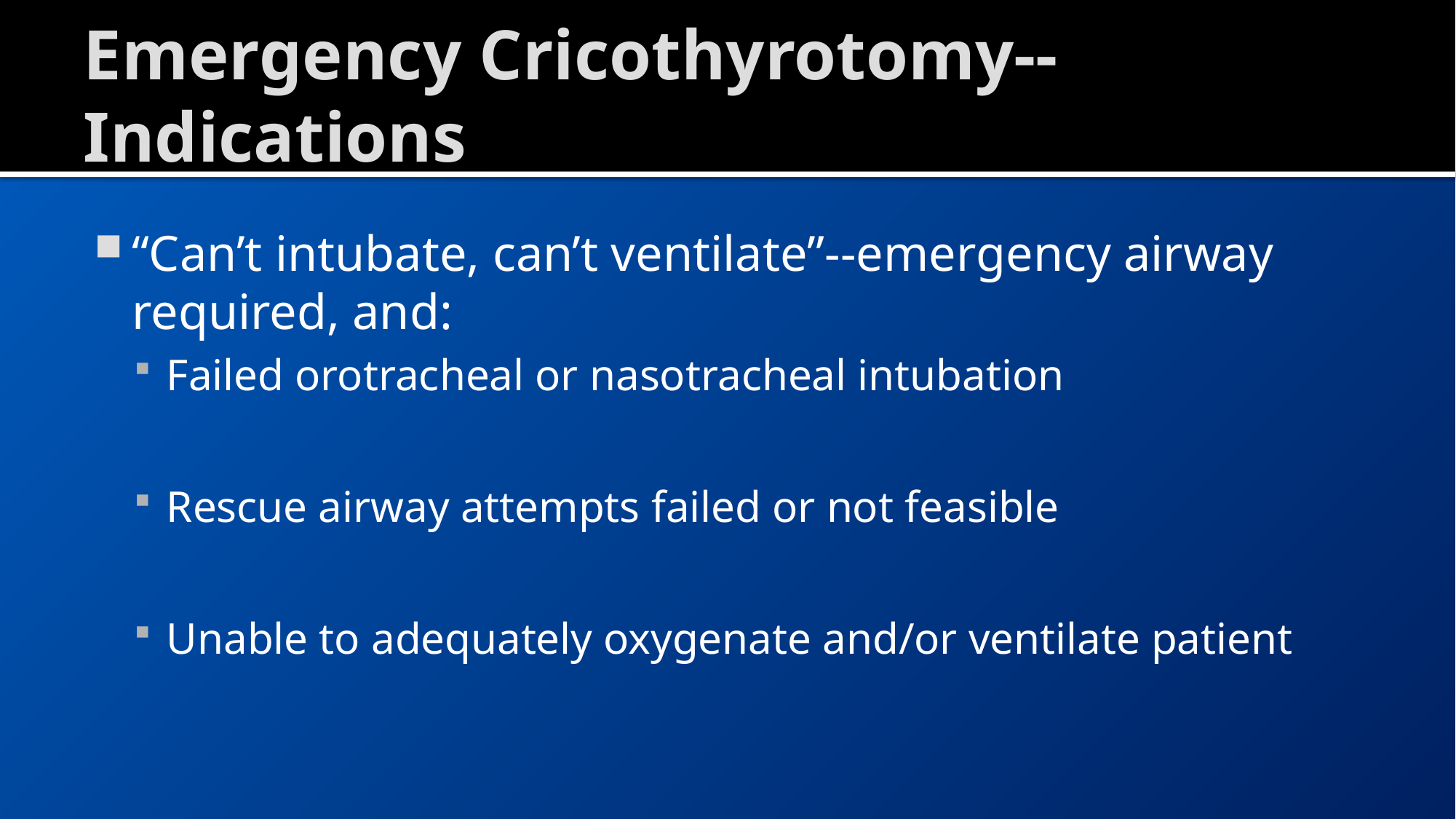

# Emergency Cricothyrotomy--Indications
“Can’t intubate, can’t ventilate”--emergency airway required, and:
Failed orotracheal or nasotracheal intubation
Rescue airway attempts failed or not feasible
Unable to adequately oxygenate and/or ventilate patient

## Slide 4
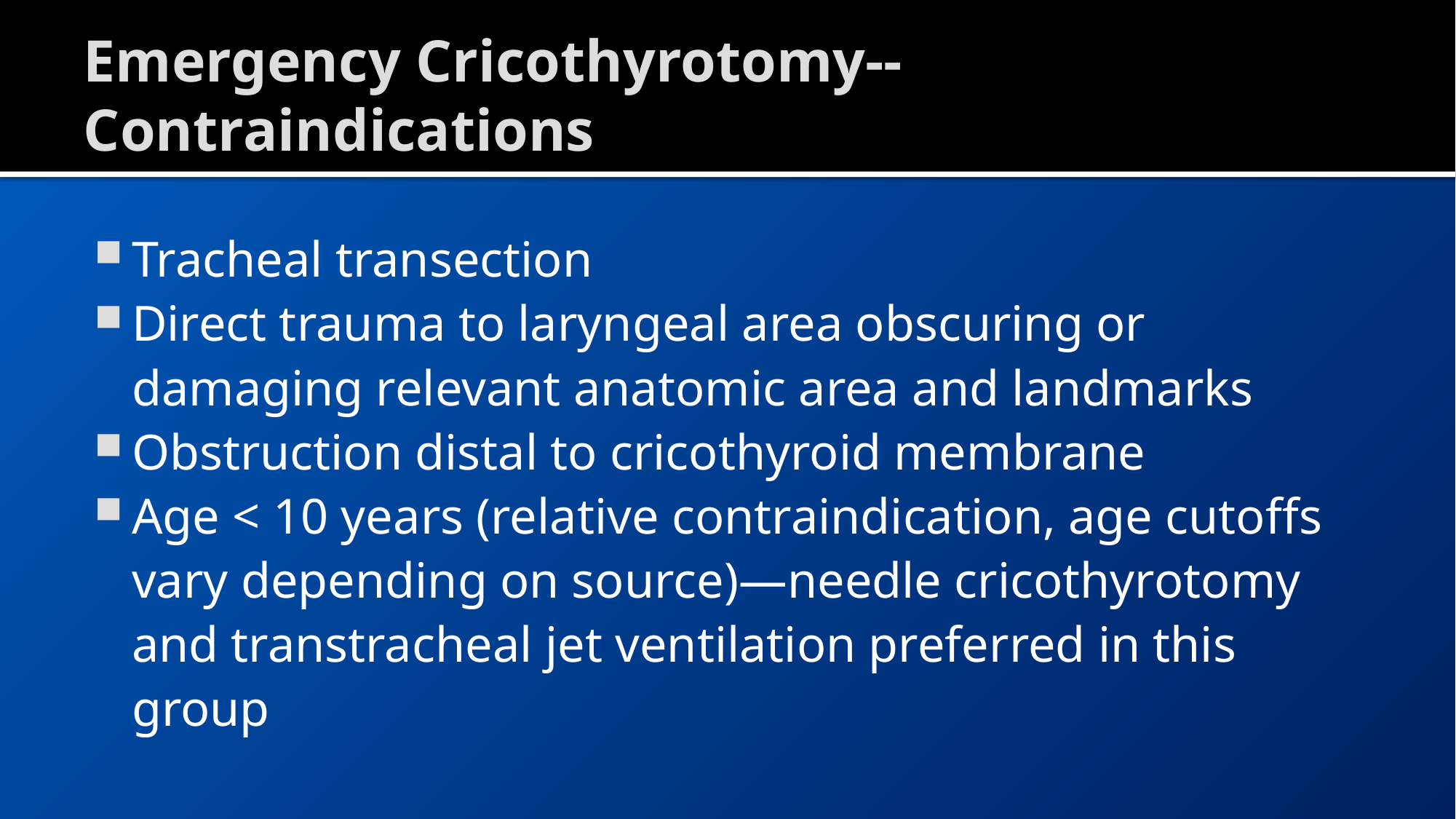

# Emergency Cricothyrotomy--Contraindications
Tracheal transection
Direct trauma to laryngeal area obscuring or damaging relevant anatomic area and landmarks
Obstruction distal to cricothyroid membrane
Age < 10 years (relative contraindication, age cutoffs vary depending on source)—needle cricothyrotomy and transtracheal jet ventilation preferred in this group

## Slide 5
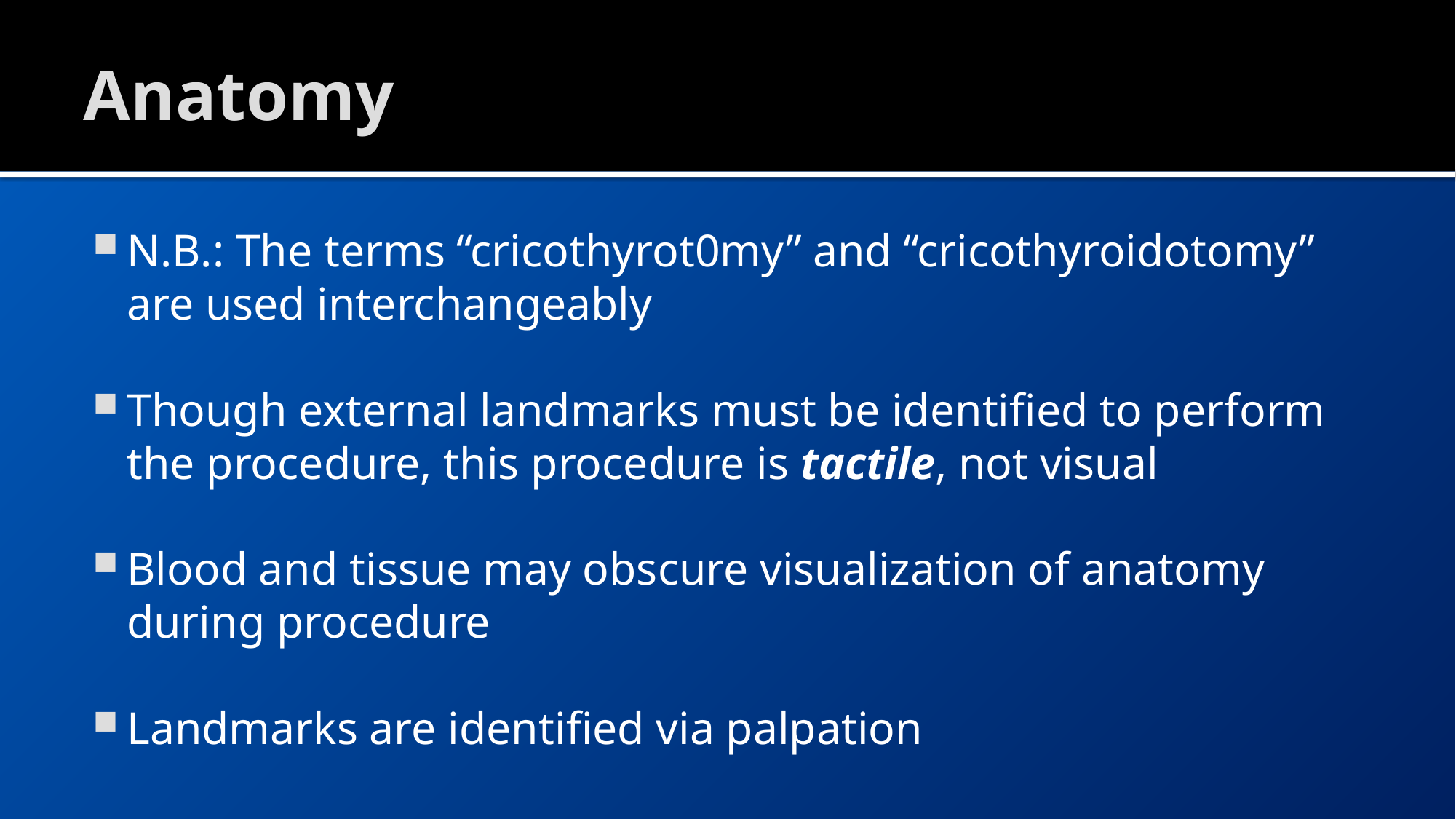

# Anatomy
N.B.: The terms “cricothyrot0my” and “cricothyroidotomy” are used interchangeably
Though external landmarks must be identified to perform the procedure, this procedure is tactile, not visual
Blood and tissue may obscure visualization of anatomy during procedure
Landmarks are identified via palpation

## Slide 6
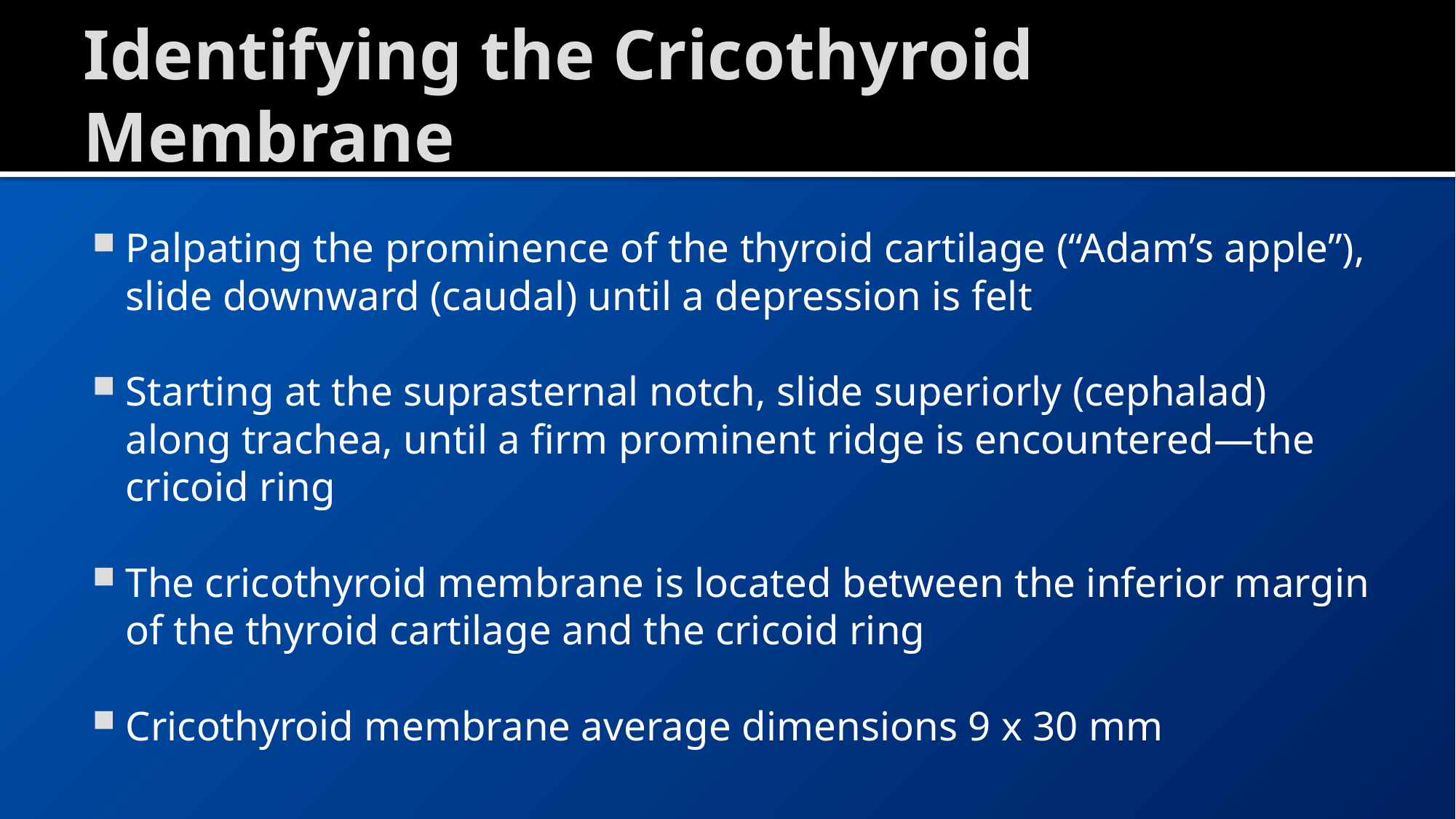

# Identifying the Cricothyroid Membrane
Palpating the prominence of the thyroid cartilage (“Adam’s apple”), slide downward (caudal) until a depression is felt
Starting at the suprasternal notch, slide superiorly (cephalad) along trachea, until a firm prominent ridge is encountered—the cricoid ring
The cricothyroid membrane is located between the inferior margin of the thyroid cartilage and the cricoid ring
Cricothyroid membrane average dimensions 9 x 30 mm

## Slide 7
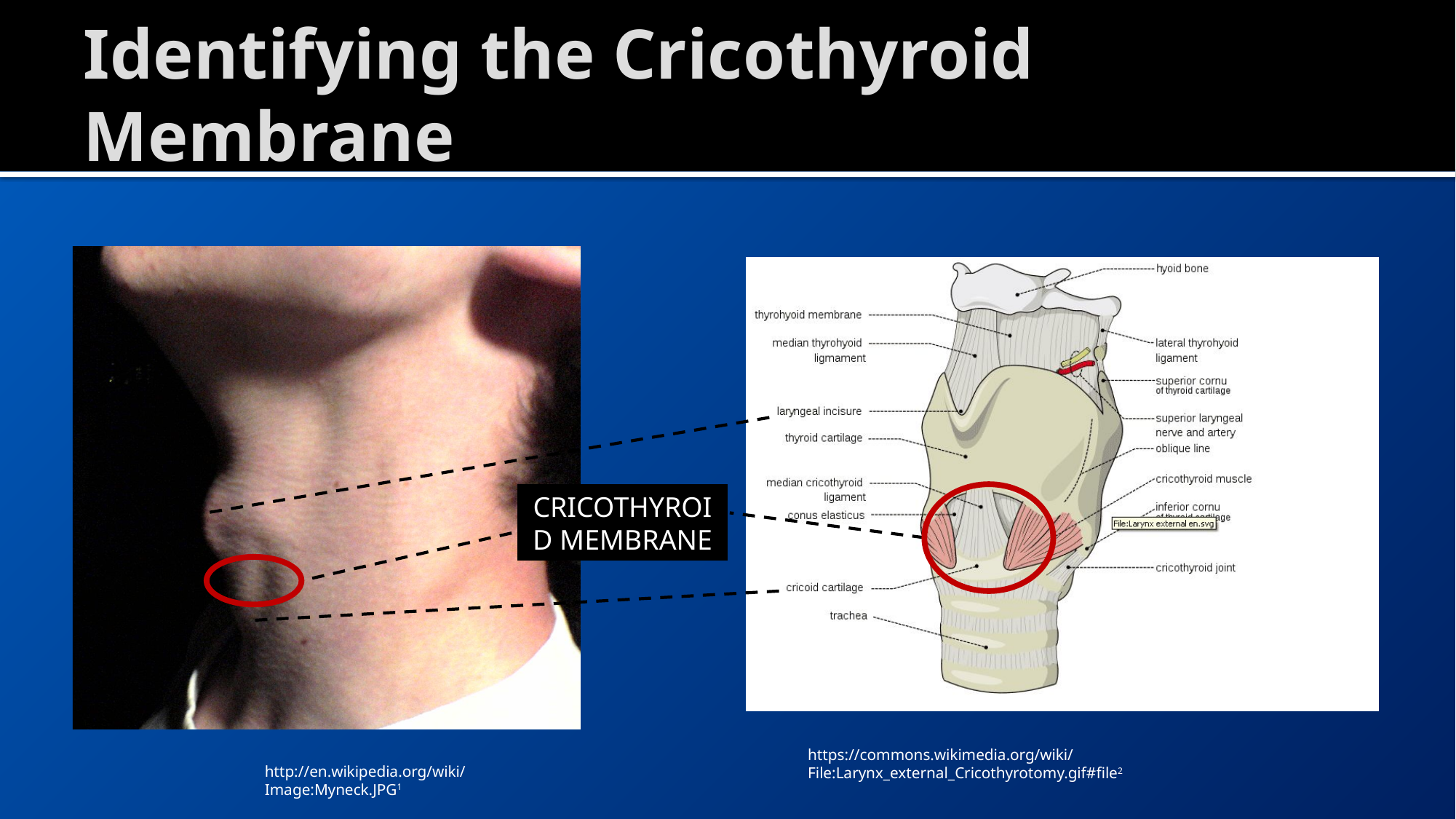

# Identifying the Cricothyroid Membrane
CRICOTHYROID MEMBRANE
https://commons.wikimedia.org/wiki/File:Larynx_external_Cricothyrotomy.gif#file2
http://en.wikipedia.org/wiki/Image:Myneck.JPG1

## Slide 8
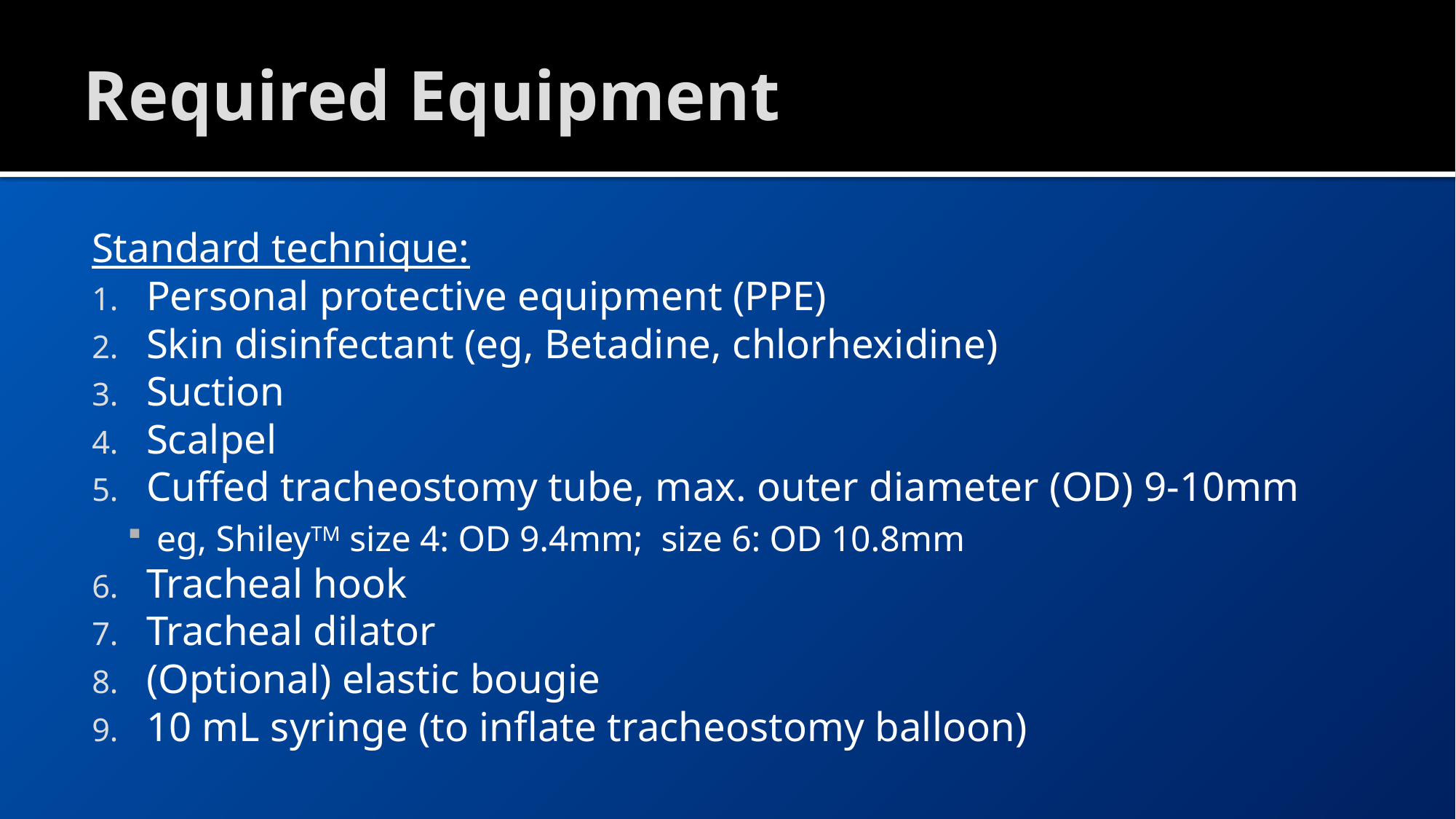

# Required Equipment
Standard technique:
Personal protective equipment (PPE)
Skin disinfectant (eg, Betadine, chlorhexidine)
Suction
Scalpel
Cuffed tracheostomy tube, max. outer diameter (OD) 9-10mm
eg, ShileyTM size 4: OD 9.4mm; size 6: OD 10.8mm
Tracheal hook
Tracheal dilator
(Optional) elastic bougie
10 mL syringe (to inflate tracheostomy balloon)

## Slide 9
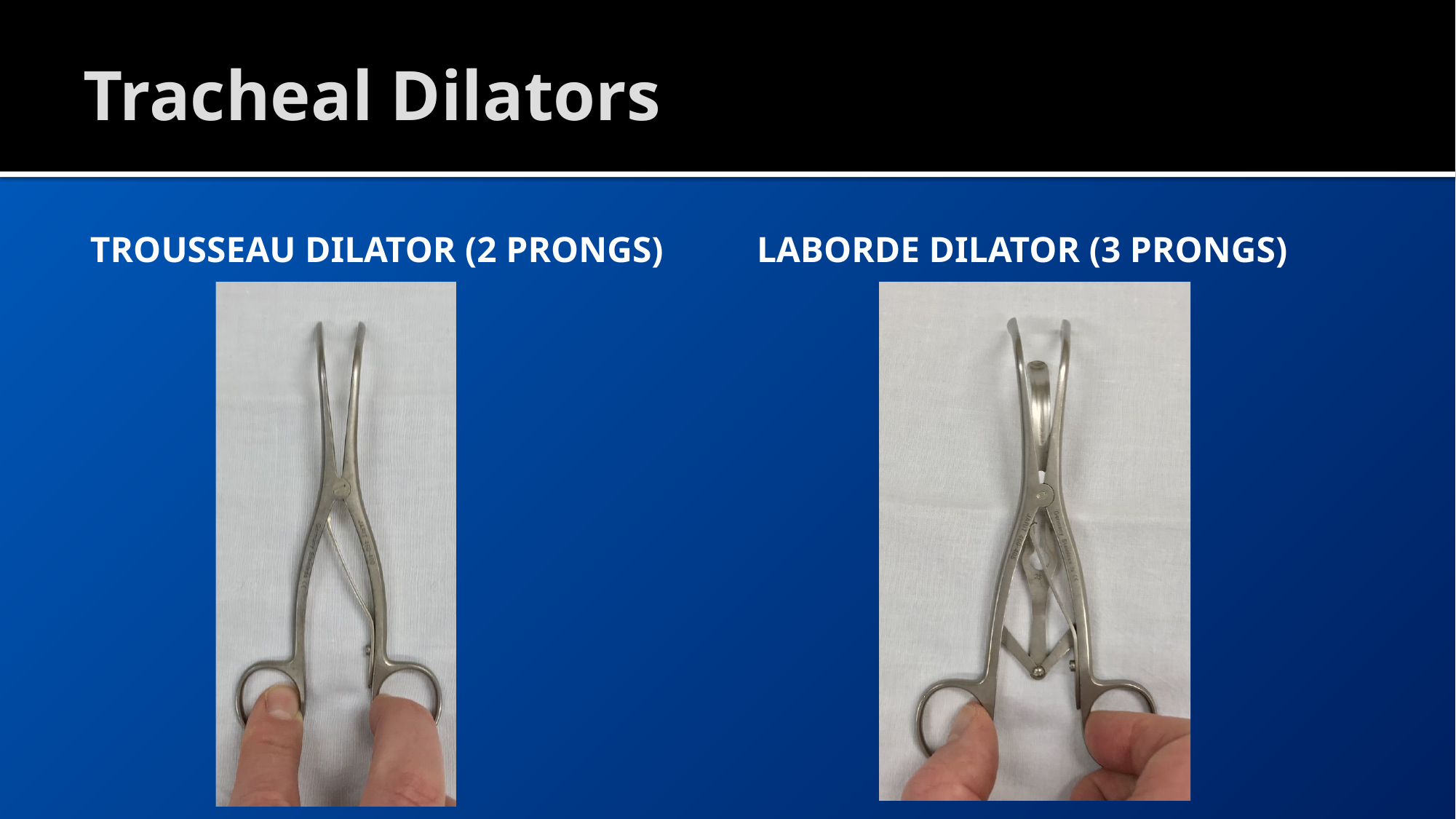

# Tracheal Dilators
Trousseau dilator (2 prongs)
Laborde dilator (3 prongs)

## Slide 10
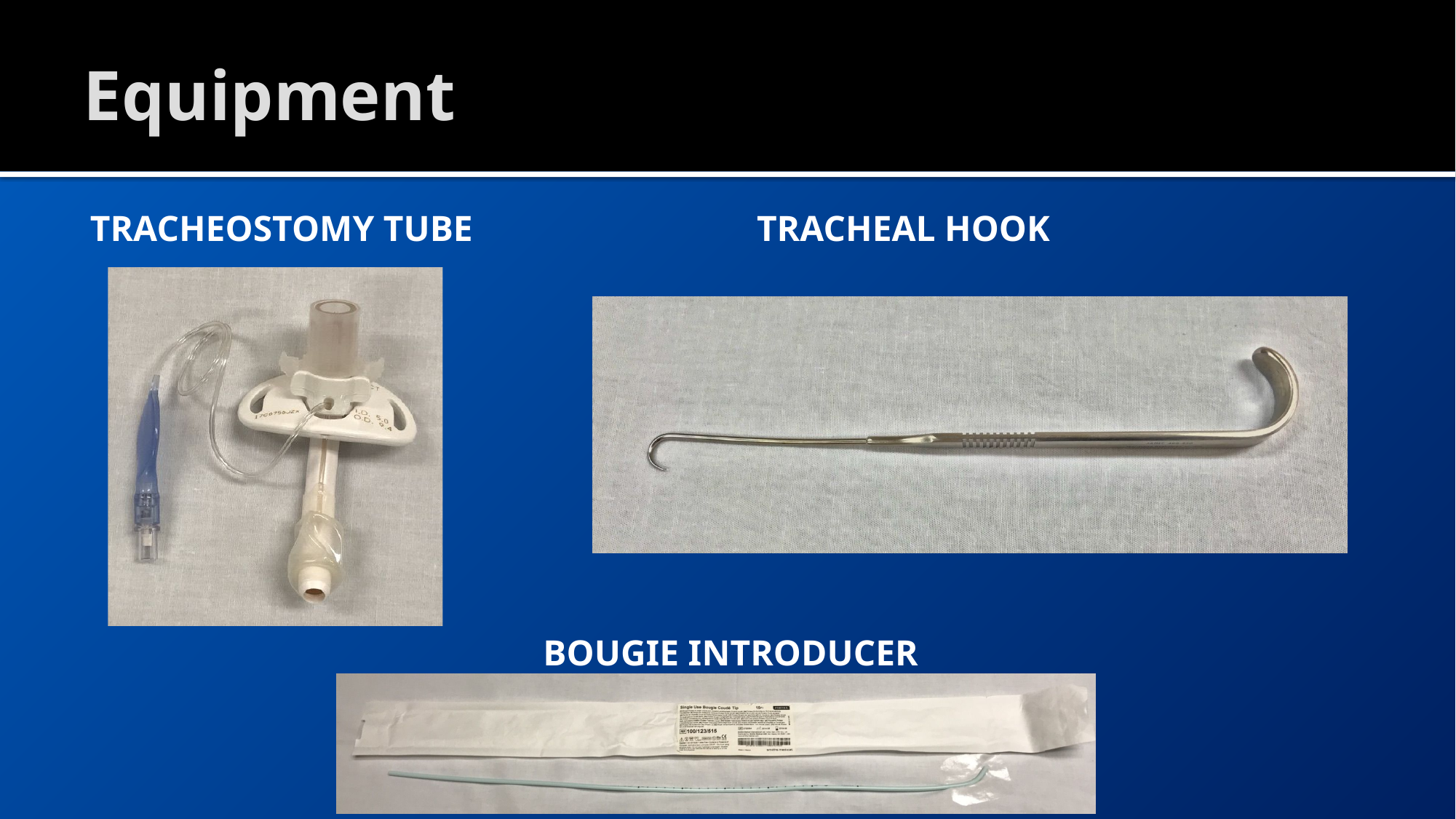

# Equipment
Tracheal hook
Tracheostomy tube
Bougie introducer

## Slide 11
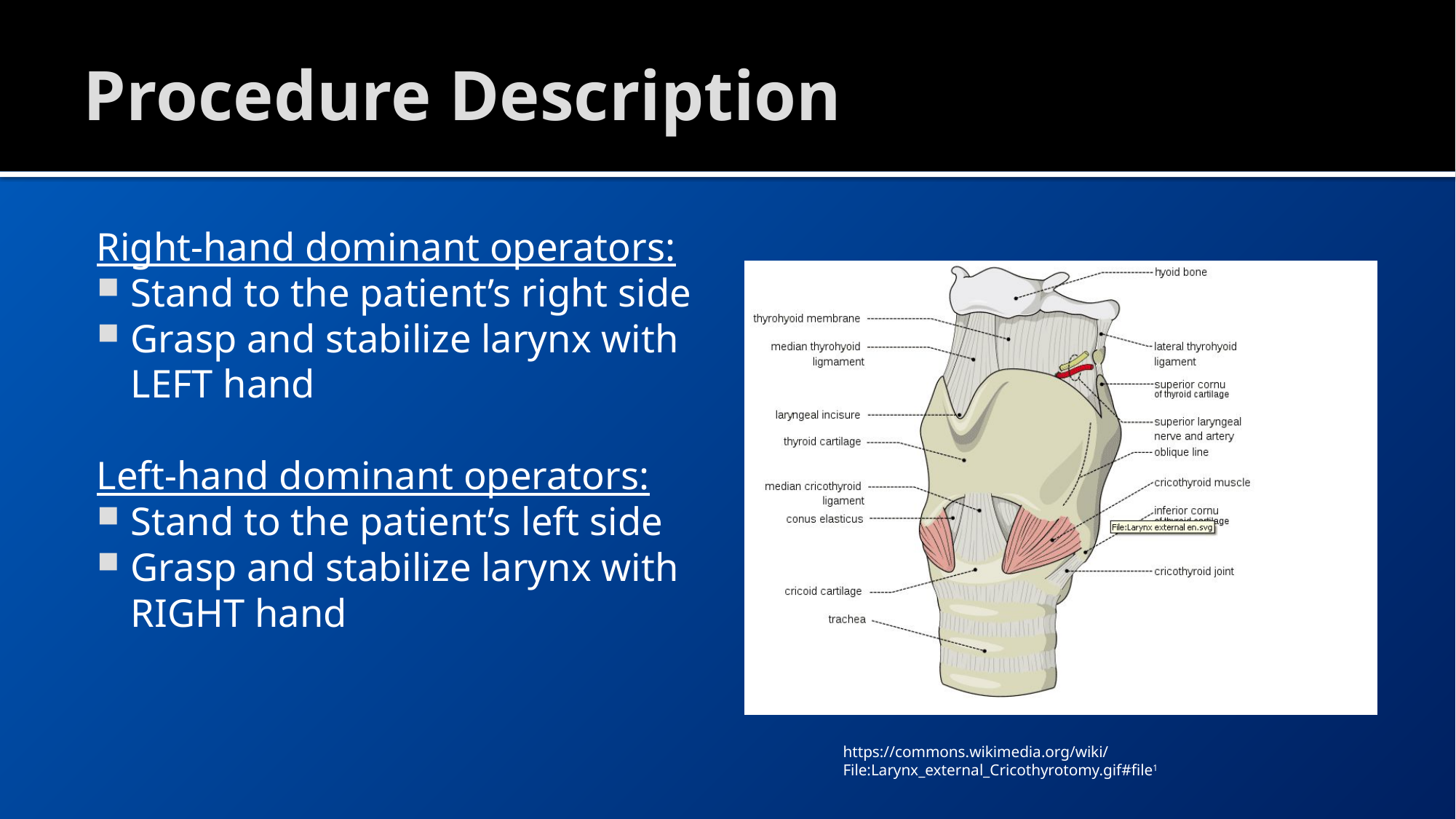

# Procedure Description
Right-hand dominant operators:
Stand to the patient’s right side
Grasp and stabilize larynx with LEFT hand
Left-hand dominant operators:
Stand to the patient’s left side
Grasp and stabilize larynx with RIGHT hand
https://commons.wikimedia.org/wiki/File:Larynx_external_Cricothyrotomy.gif#file1

## Slide 12
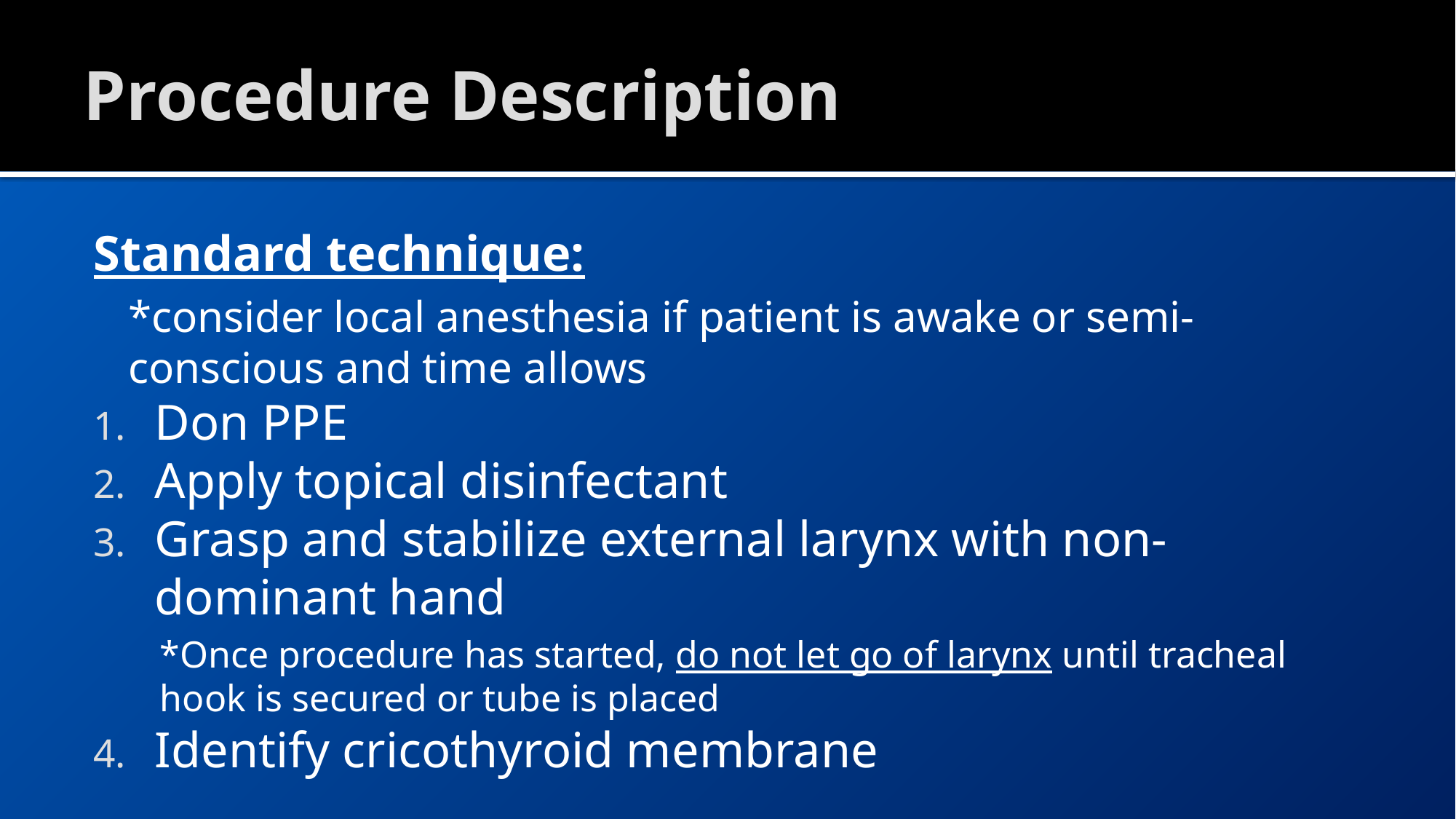

# Procedure Description
Standard technique:
*consider local anesthesia if patient is awake or semi-conscious and time allows
Don PPE
Apply topical disinfectant
Grasp and stabilize external larynx with non-dominant hand
*Once procedure has started, do not let go of larynx until tracheal hook is secured or tube is placed
Identify cricothyroid membrane

## Slide 13
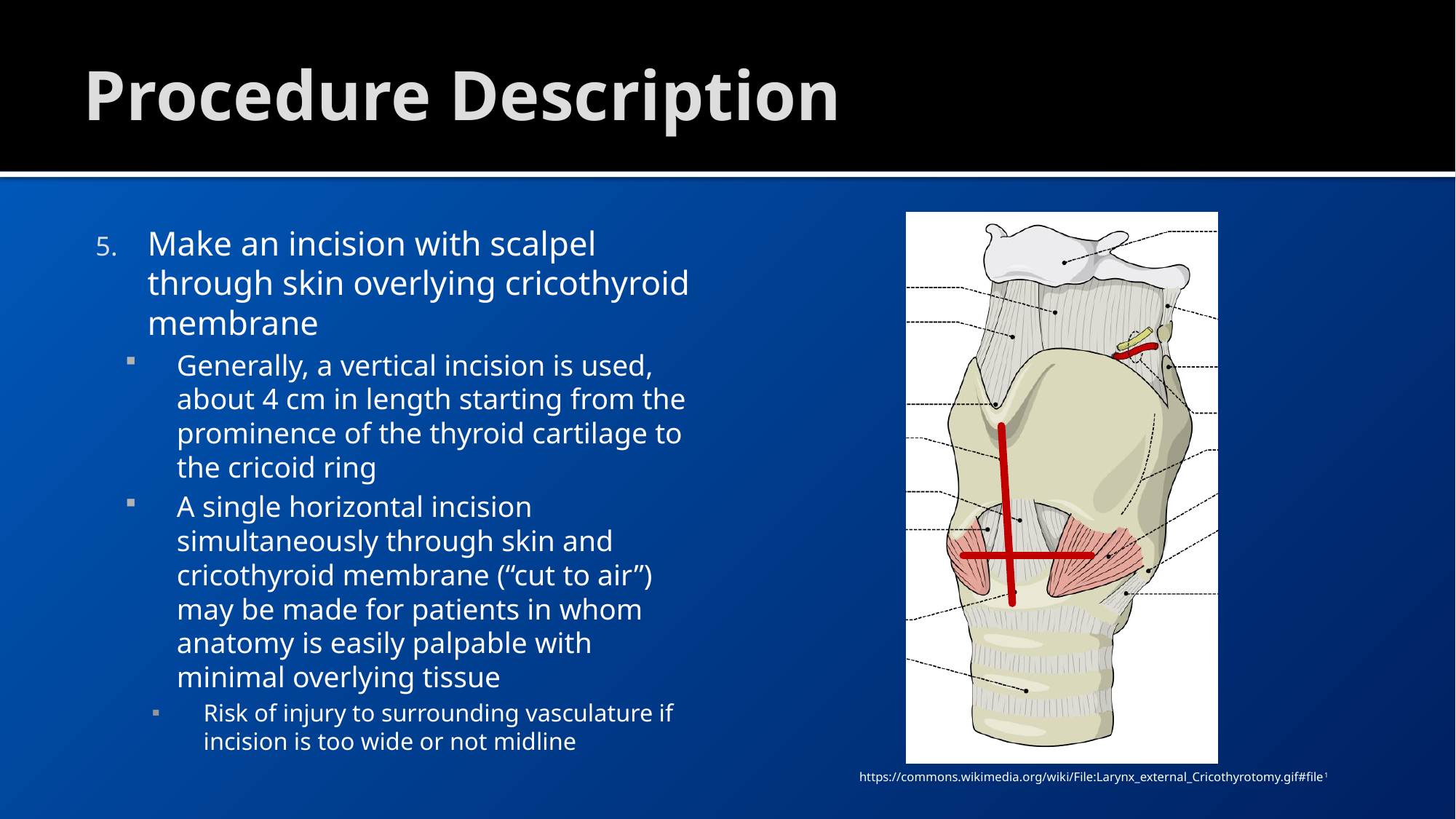

# Procedure Description
Make an incision with scalpel through skin overlying cricothyroid membrane
Generally, a vertical incision is used, about 4 cm in length starting from the prominence of the thyroid cartilage to the cricoid ring
A single horizontal incision simultaneously through skin and cricothyroid membrane (“cut to air”) may be made for patients in whom anatomy is easily palpable with minimal overlying tissue
Risk of injury to surrounding vasculature if incision is too wide or not midline
https://commons.wikimedia.org/wiki/File:Larynx_external_Cricothyrotomy.gif#file1

## Slide 14
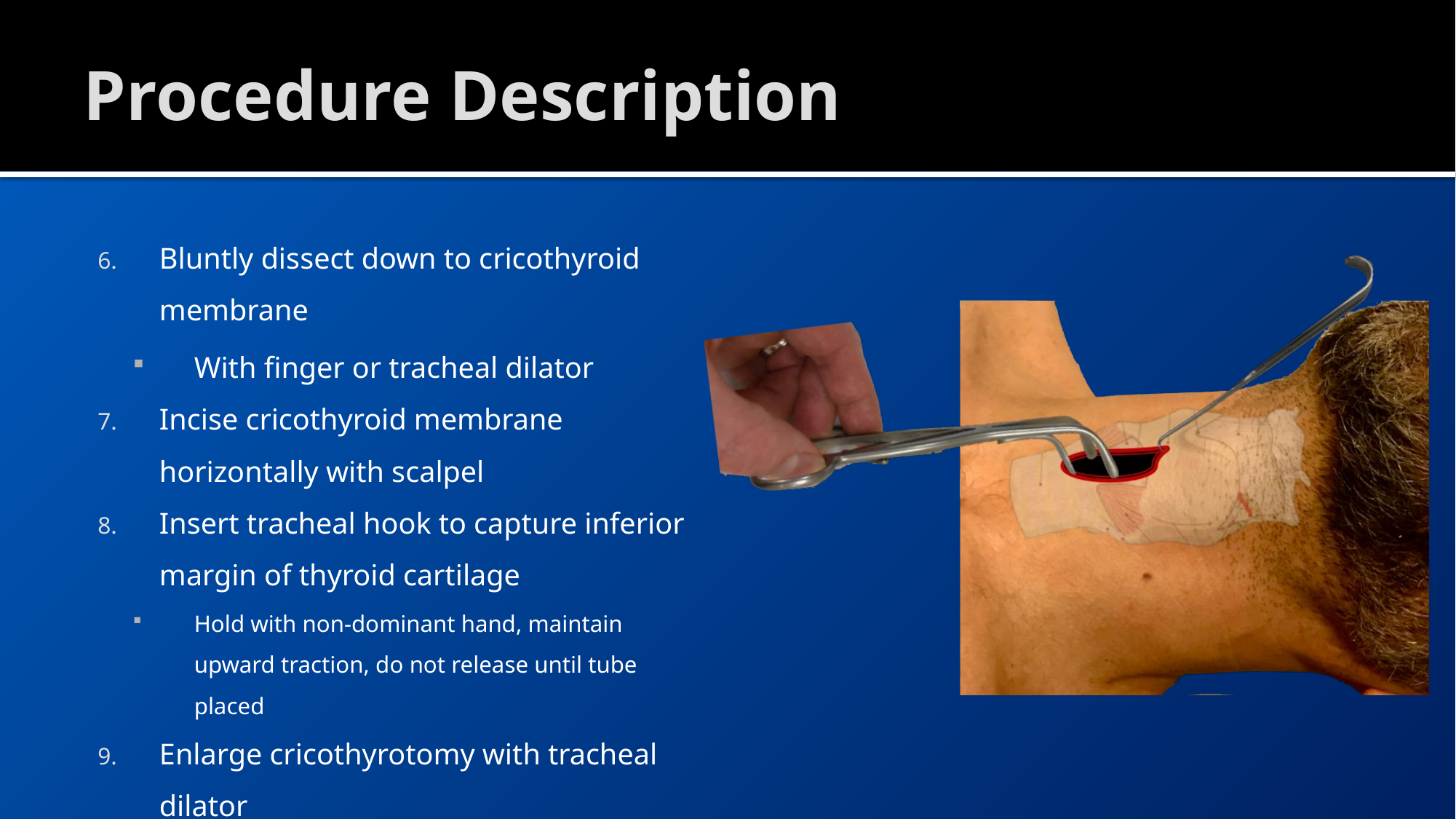

# Procedure Description
Bluntly dissect down to cricothyroid membrane
With finger or tracheal dilator
Incise cricothyroid membrane horizontally with scalpel
Insert tracheal hook to capture inferior margin of thyroid cartilage
Hold with non-dominant hand, maintain upward traction, do not release until tube placed
Enlarge cricothyrotomy with tracheal dilator

## Slide 15
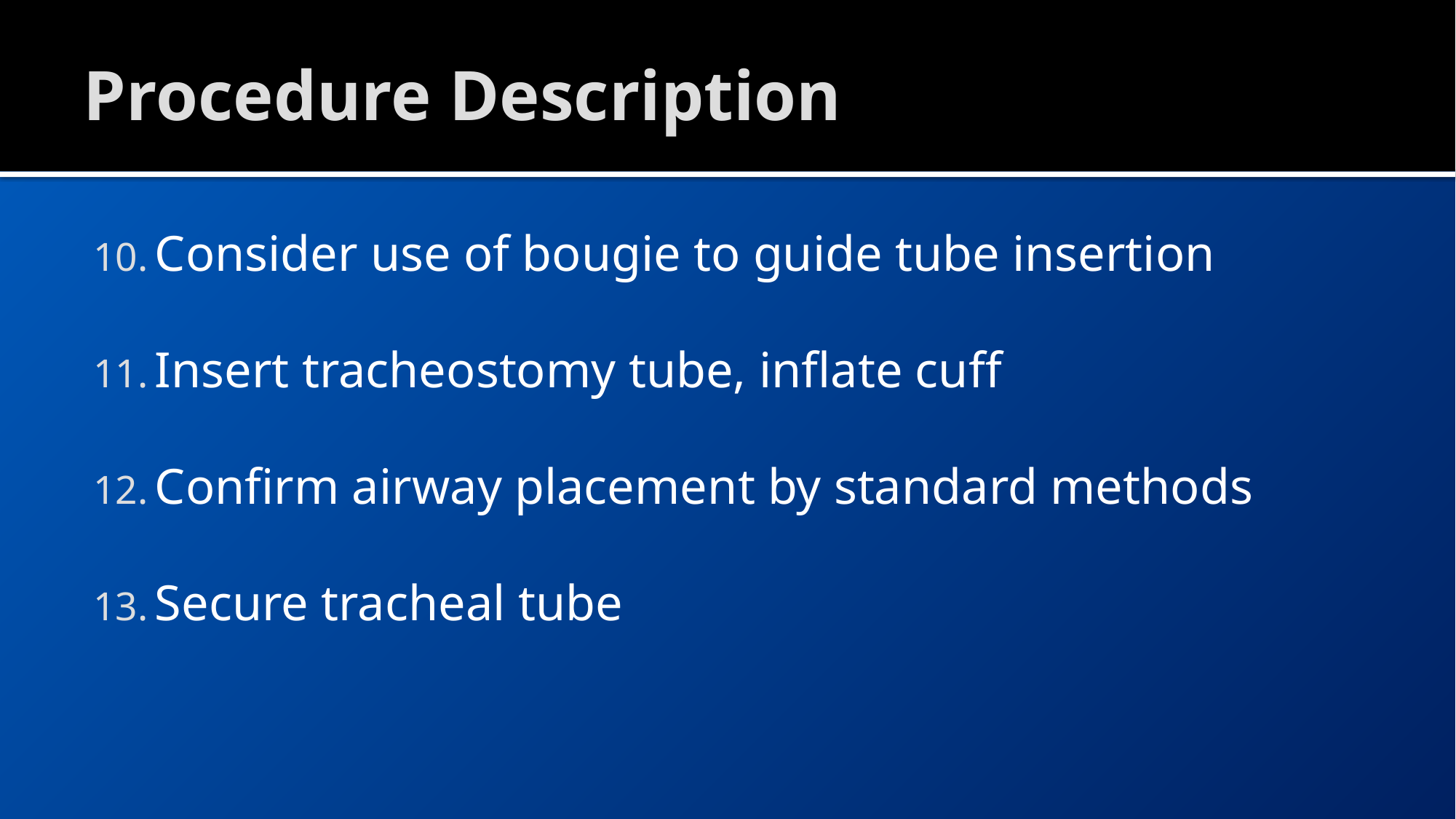

# Procedure Description
Consider use of bougie to guide tube insertion
Insert tracheostomy tube, inflate cuff
Confirm airway placement by standard methods
Secure tracheal tube

## Slide 16
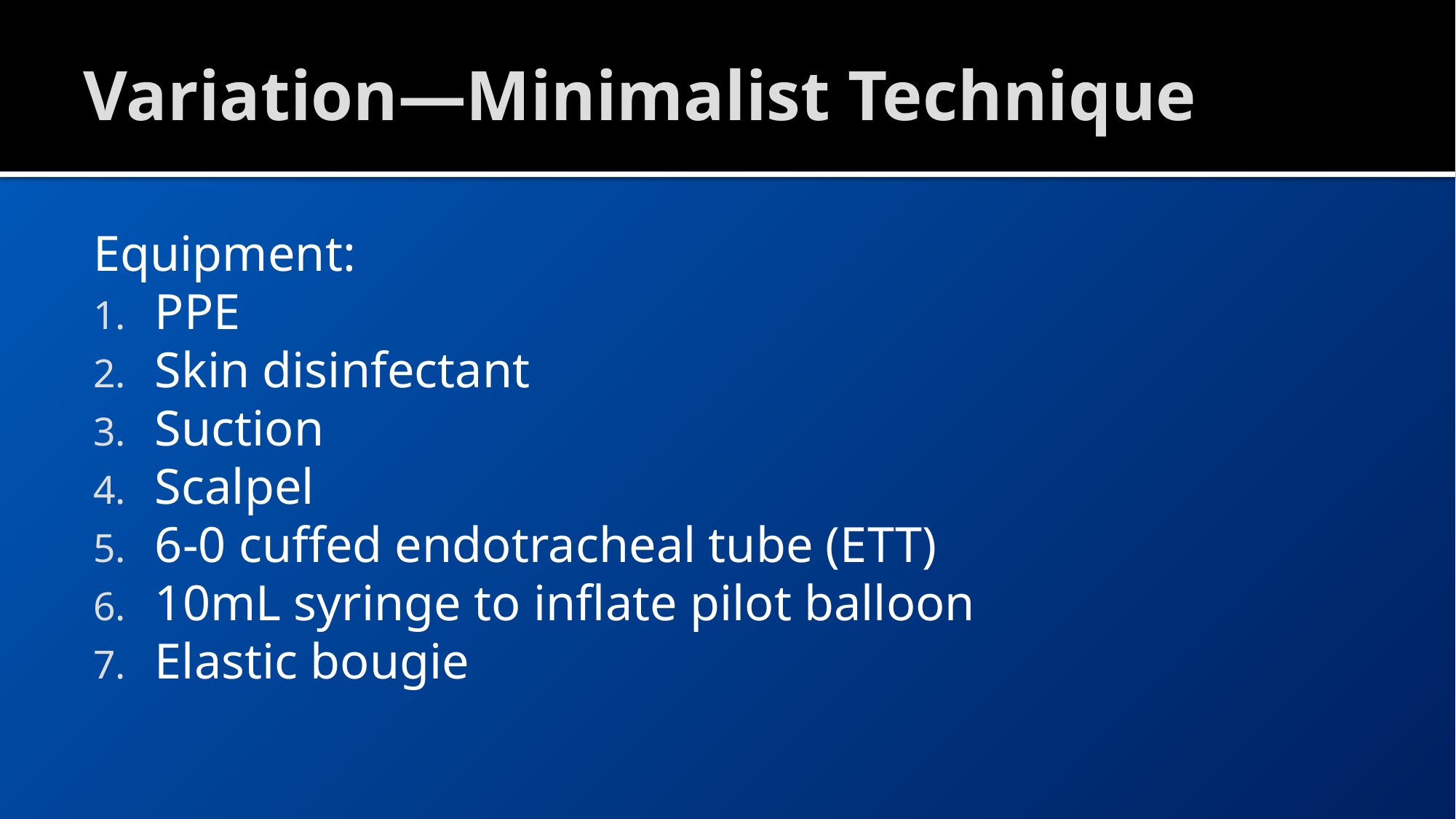

# Variation—Minimalist Technique
Equipment:
PPE
Skin disinfectant
Suction
Scalpel
6-0 cuffed endotracheal tube (ETT)
10mL syringe to inflate pilot balloon
Elastic bougie

## Slide 17
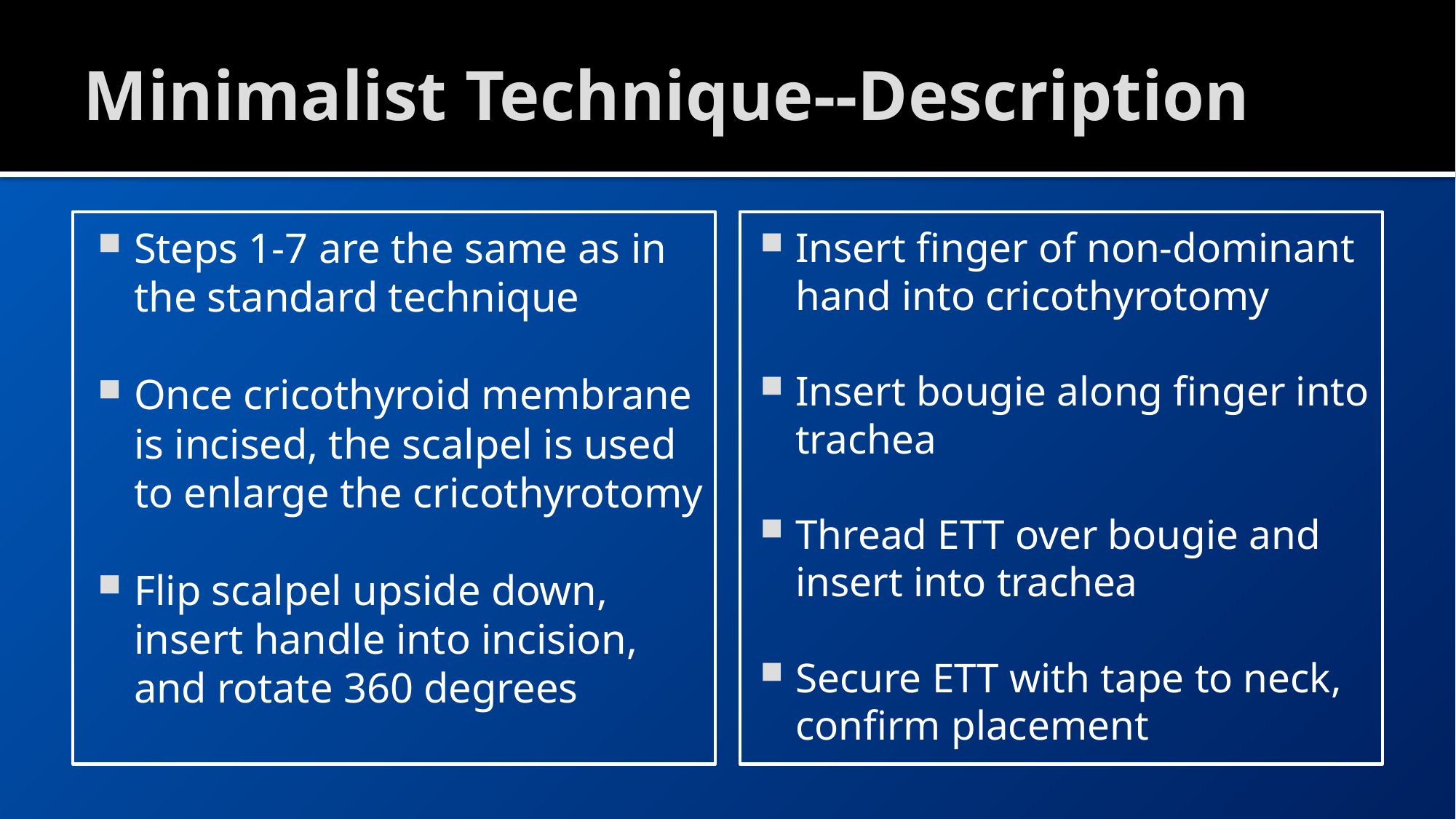

# Minimalist Technique--Description
Steps 1-7 are the same as in the standard technique
Once cricothyroid membrane is incised, the scalpel is used to enlarge the cricothyrotomy
Flip scalpel upside down, insert handle into incision, and rotate 360 degrees
Insert finger of non-dominant hand into cricothyrotomy
Insert bougie along finger into trachea
Thread ETT over bougie and insert into trachea
Secure ETT with tape to neck, confirm placement

## Slide 18
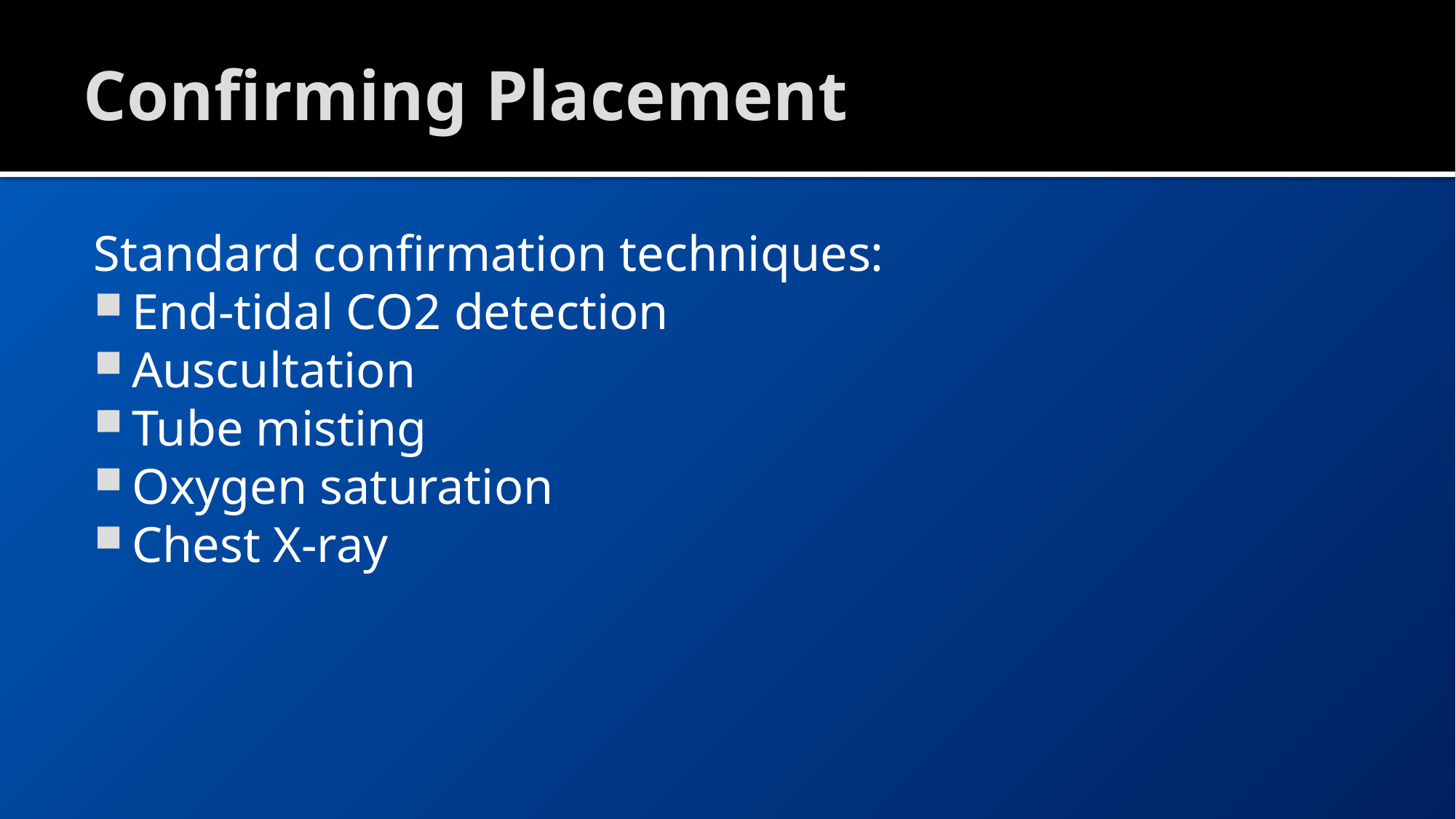

# Confirming Placement
Standard confirmation techniques:
End-tidal CO2 detection
Auscultation
Tube misting
Oxygen saturation
Chest X-ray

## Slide 19
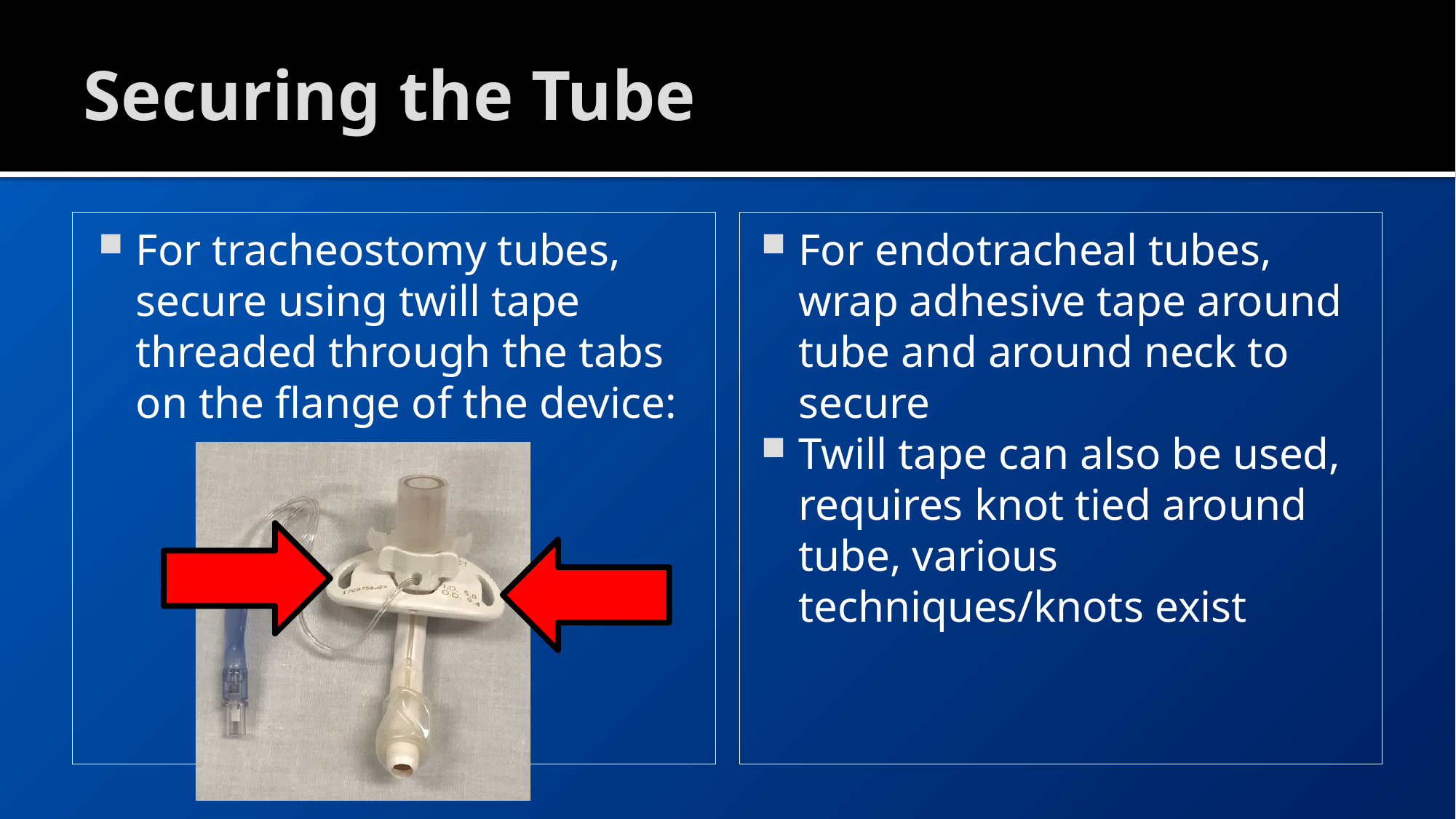

# Securing the Tube
For tracheostomy tubes, secure using twill tape threaded through the tabs on the flange of the device:
For endotracheal tubes, wrap adhesive tape around tube and around neck to secure
Twill tape can also be used, requires knot tied around tube, various techniques/knots exist

## Slide 20
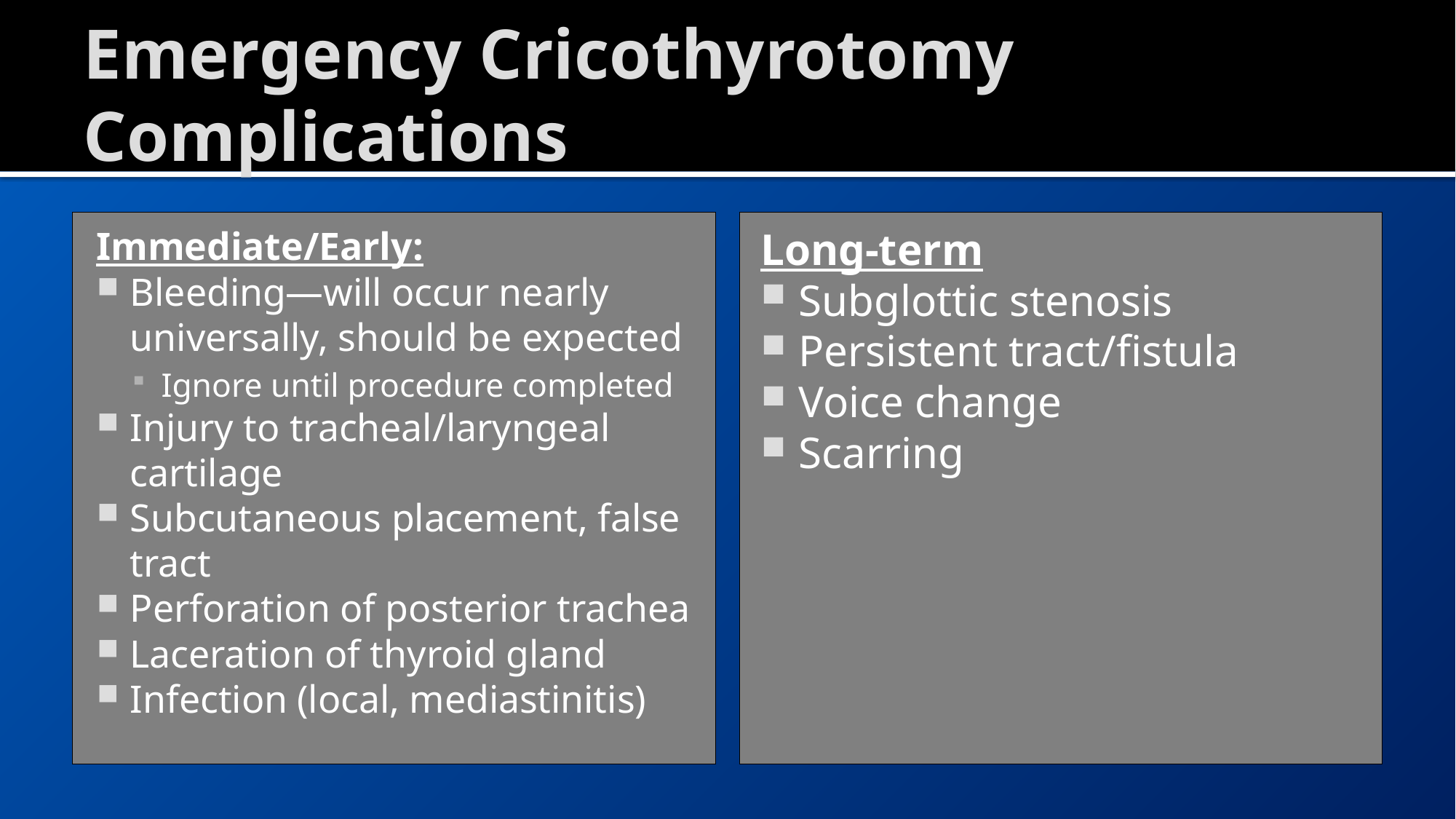

# Emergency Cricothyrotomy Complications
Immediate/Early:
Bleeding—will occur nearly universally, should be expected
Ignore until procedure completed
Injury to tracheal/laryngeal cartilage
Subcutaneous placement, false tract
Perforation of posterior trachea
Laceration of thyroid gland
Infection (local, mediastinitis)
Long-term
Subglottic stenosis
Persistent tract/fistula
Voice change
Scarring
